# Scalable Synthesis of All Stereoisomers of 2-Aminocyclopentanecarboxylic Acid—A Toolbox for Peptide Foldamer Chemistry

**DOI:** 10.1021/acs.joc.3c02991

**Published:** 2024-03-28

**Authors:** Vitaly Kovalenko, Ewa Rudzińska-Szostak, Katarzyna Ślepokura, Łukasz Berlicki

**Affiliations:** † Department of Bioorganic Chemistry, Wrocław University of Science and Technology, Wyb. Wyspiańskiego 27, 50-370 Wrocław, Poland; ‡ Faculty of Chemistry, University of Wrocław, F. Joliot-Curie 14, 50-383 Wrocław, Poland

## Abstract

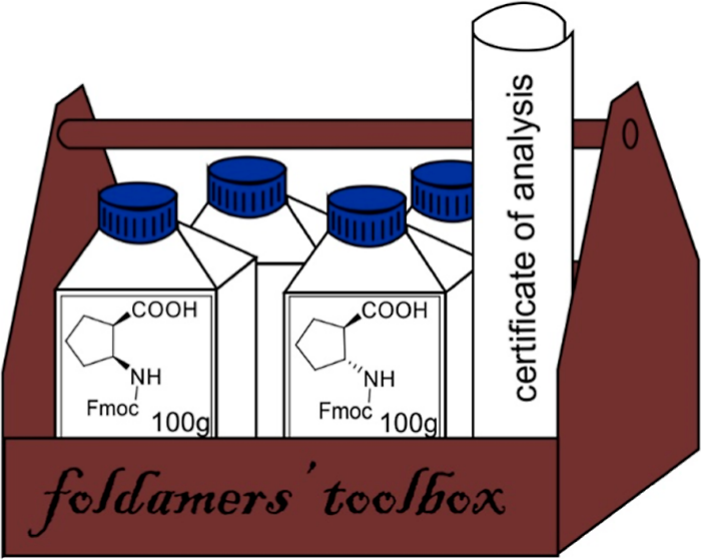

Although the construction of peptides with well-defined three-dimensional structures and predictable functions, including biological activity, using conformationally constrained β-amino acids has been shown to be a very successful strategy, their broad application is limited by access to the appropriate building blocks. In particular, *trans*- and *cis*-stereoisomers of 2-aminocyclopentanecarboxylic acid (ACPC) are of high interest. The scalable synthesis of all four stereoisomers of Fmoc derivatives of ACPC is presented with NMR-based analysis methods for their enantiomeric purity.

## Introduction

The field of foldamers, defined as oligomers with a high propensity to fold in solution, was established over thirty years ago by the seminal works of Gellman and Seebach.^1−3^ Although the definition of this research area is very broad, major achievements were made for peptides incorporating noncanonical amino acid residues.^4^ Several investigations concerned structural studies that revealed the formation of diversified secondary structures, including various types of helices and extended structures.^5,6^ In particular, conformationally constrained β-amino acid building blocks have been used successfully.^7^ Depending on the structure of the constraining fragment and the sequence pattern, different folding preferences were observed, which were finally described in stereochemical patterning theory.^8^ Recently, tertiary structures of β-amino acid-containing peptide foldamers were reported.^9−12^ It is worth mentioning that 2-aminocyclopentanecarboxylic acid (ACPC) was one of the preferred building blocks due to the combination of high conformational stability with compatibility to standard conditions of solid-phase peptide synthesis.^13^

Peptide foldamers incorporating β-amino acids, such as ACPC, have shown numerous unique properties and activities.^14,15^ Excellent biological activities ranging from anticancer, antibacterial, and antiviral to antifungal activities, combined with high resistance to proteolytic degradation, make members of this class of compounds valuable drug candidates.^16−21^ Moreover, there are peptide foldamers with interesting catalytic activity.^22−25^ Another interesting property of this class of compounds is the possibility of the formation of nanostructures.^26,27^

Considering all of the achievements mentioned above, it could be stated without doubt that molecules with well-predictable three-dimensional structures and chosen functions can be constructed using constrained β-amino acid building blocks. However, the major technical problem with the wide application of this technology is access to appropriate amounts of enantiomerically pure derivatives of constrained β-amino acids useful for solid-phase peptide synthesis, in particular, ACPC. Although synthetic routes leading to Fmoc-protected ACPC are known, they cannot be safely scaled up to reach all four stereoisomers in multidecagram quantities. Herein, we report a scalable and reliable scheme that allows us to obtain either cis or trans isomers in enantiopure form. Common precursors and only readily available reagents were used. We also elaborate on the simple and reliable method of analysis of the enantiomeric purity of Fmoc-ACPC stereoisomers that is based on ^1^H NMR spectra of the analyte with a chiral solvating agent (CSA).

## Results and Discussion

**Figure 1:**
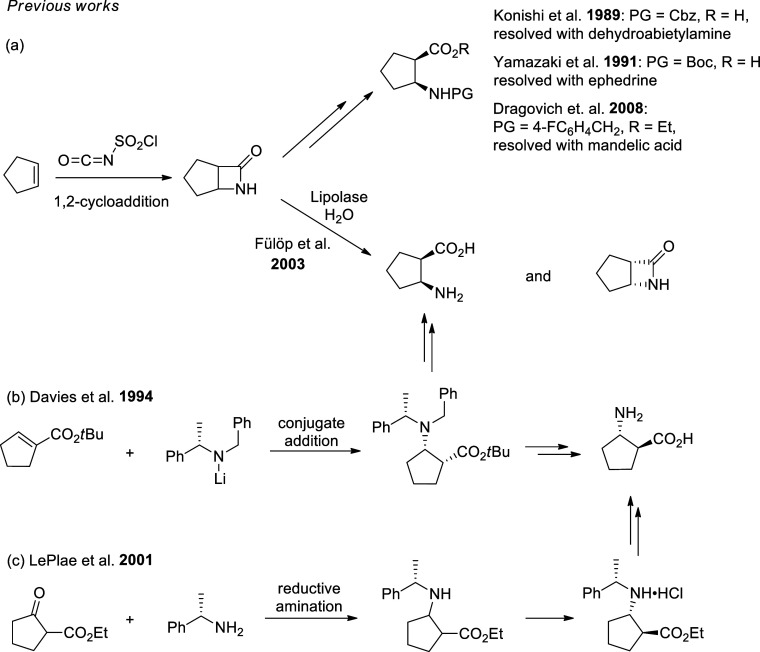
Summary of previous works on ACPC synthesis which used lactam hydrolysis (a), conjugate addition (b), or reductive amination (c).

Based on the literature data, we identified three main strategies dedicated to the synthesis of enantiopure *cis*- and *trans*-ACPC (Figure 1). In the first approach, bicyclic β-lactam (Figure 1a), a racemic precursor of *cis*-ACPC accessible by the 1,2-dipolar cycloaddition of chlorosulfonyl isocyanate to cyclopentene, is used. Fülöp et al. directly converted this lactam into a chiral amino acid by enzymatic hydrolysis.^28^ Later, the same research group developed enzymatic kinetic resolution for the amide and ester of *cis*-ACPC.^29,30^ The crystallization-based resolution methods can be an attractive alternative to the enzymatic approach. For example, *N*-Cbz- and *N*-Boc-protected *cis*-ACPC were resolved with dehydroabiethylamine^31^ and ephedrine,^32^ respectively, while the ethyl ester of *N*-4-fluorobenzylated *cis*-ACPC was resolved with mandelic acid.^33^ The different approach to ACPC was developed by Davies and co-workers^34^ by applying conjugate addition of α-phenylethylamine-derived lithium amide to *tert*-butyl cyclopentene-1-carboxylate (Figure 1b). It was shown that the initially formed *cis*-adduct can be epimerized into a *trans*-isomer. This methodology is flexible toward all possible stereoisomers of ACPC; however, it requires a careful process for the conjugate addition reaction at low temperature and subsequent chromatographic separations. The strategy proposed by LePlae and co-workers (Figure 1c)^35^ is based on reductive amination of the 2-oxocyclopentane carboxylic acid ethyl ester with α-phenylethylamine, and the product of this step crystallized as a single stereoisomer in the form of a hydrochloride salt.

**Scheme 1:**
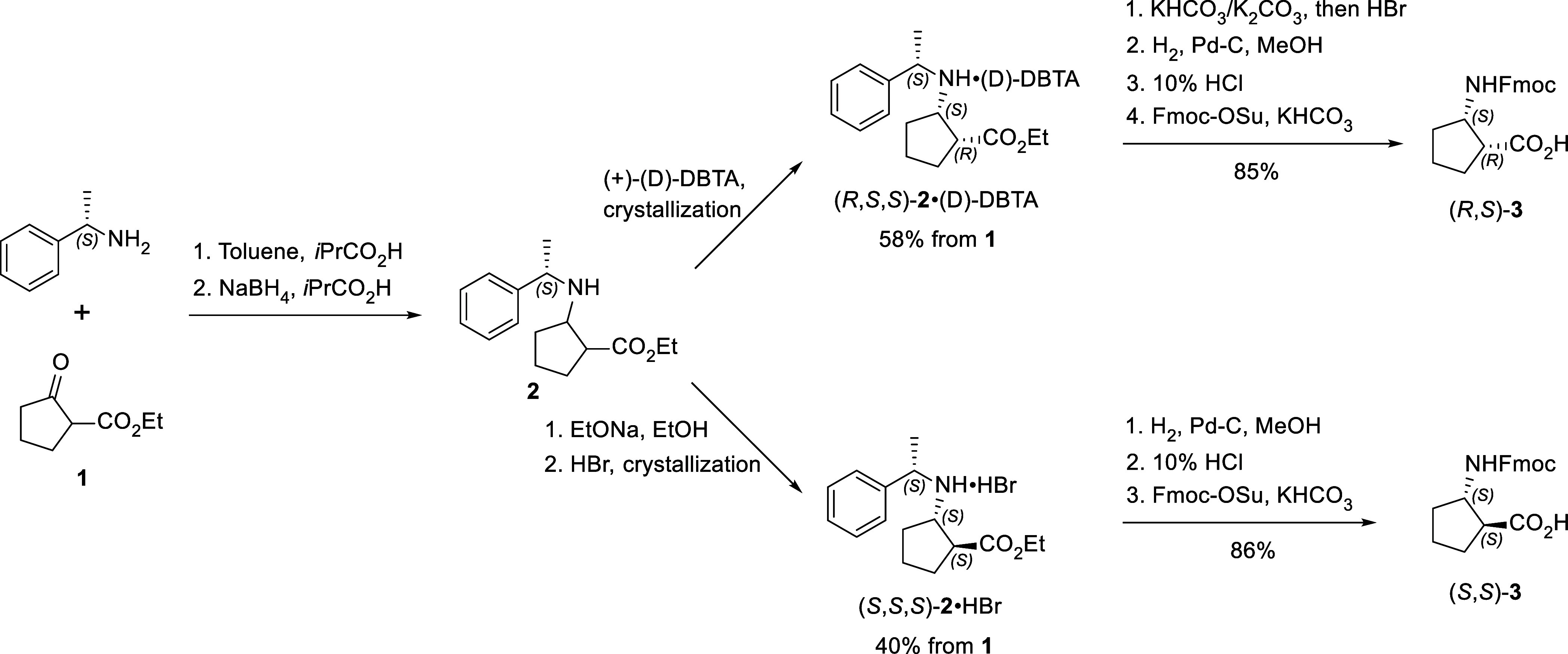
Synthetic Pathway to Enantiomerically Pure Fmoc Derivatives of *cis*- and *trans*-ACPC

The LePlae synthesis can be adapted to both stereoisomers of *trans*-ACPC but, unfortunately, not to *cis* stereoisomers. When it comes to scale-up, this eventually leads to an increase in the load of toxic cyanoborohydride, which is needed for reductive amination. Therefore, our objective was to address both of these shortcomings. Initial attempts to perform the reductive amination step using NaBH_4_ in EtOH as a reductive agent at room or elevated temperature were unsuccessful. However, complete conversion of ketoester **1** and (*S*)-α-phenylethylamine into amino ester **2** was achieved by using azeotropic distillation with toluene followed by reduction with NaBH_4_ in isobutyric acid (Scheme 1). A combination of these reagents was previously used for reductive amination of a similar 2-oxocyclohexane carboxylate.^36^ Then, following the LePlae procedure,^35^ we converted crude amino ester **2** into hydrochloride salt, but no crystallization was observed. Treatment of the crude amino ester **2** with sodium ethoxide in ethanol led to the expected epimerization of the stereocenter at the α-position. According to NMR, the initial ratio of diastereomers 1.0:0.15:0.06:0.02 shifted to the ratio 0.21:0.02:1.0:0.15 in favor of the *trans*-isomer. The best yield and purity were achieved when the reaction with EtONa was performed at 30–35 °C overnight. Increasing the temperature or using other strong bases did not improve the *cis*/*trans* ratio. The crude isomerization product gave a crystalline hydrobromide salt. Repeated crystallization of this salt from acetonitrile provided pure hydrobromide of (*S*,*S*,*S*)-**2** in the overall yield of up to 40% calculated from ketoester **1**. After the fourth crystallization, the signals of the diastereomeric impurities could not be detected by ^1^H NMR.

As the attempt to isolate pure *cis*-amino ester **2** in the same manner as *trans*-isomer **2** was unsuccessful, we investigated the formation of the salts of amino ester **2** with different organic and inorganic acids, including HCl, HBr, H_2_SO_4_ (1 and 0.5 equiv), H_2_[ZnCl_4_] (1 and 0.5 equiv), TsOH, d- and l-tartaric acid and their dibenzoyl derivatives, l-malic acid, (1*R*)- and (1*S*)-camphor sulfonic acids, oxalic acid, phthalic acid, 4-nitrobenzoic acid, and 3,5-dinitrobenzoic acid. We found that crystallization with (+)-dibenzoyl-d-tartaric acid ((D)-DBTA) efficiently removed all impurities and gave the salt of (*R*,*S*,*S*)-amino ester **2** in a 58% yield from ketoester **1** (Scheme 1). Typically, two or three crystallizations from acetonitrile were enough to obtain a pure product, but in the series of experiments, we noticed significant deviations in the yield. We concluded that prolonged heating needed to dissolve the crude solid material (especially on a large scale) affected the yield of the recrystallized material. To avoid this, a mixture of acetonitrile and water was used as a solvent for the second and third crystallizations. The addition of water to acetonitrile increased the solubility and facilitated the dissolution of solids, while further increasing the concentration of water promoted the precipitation of the salt (*R*,*S*,*S*)-**2**•(D)-DBTA from saturated solution.

**Figure 2:**
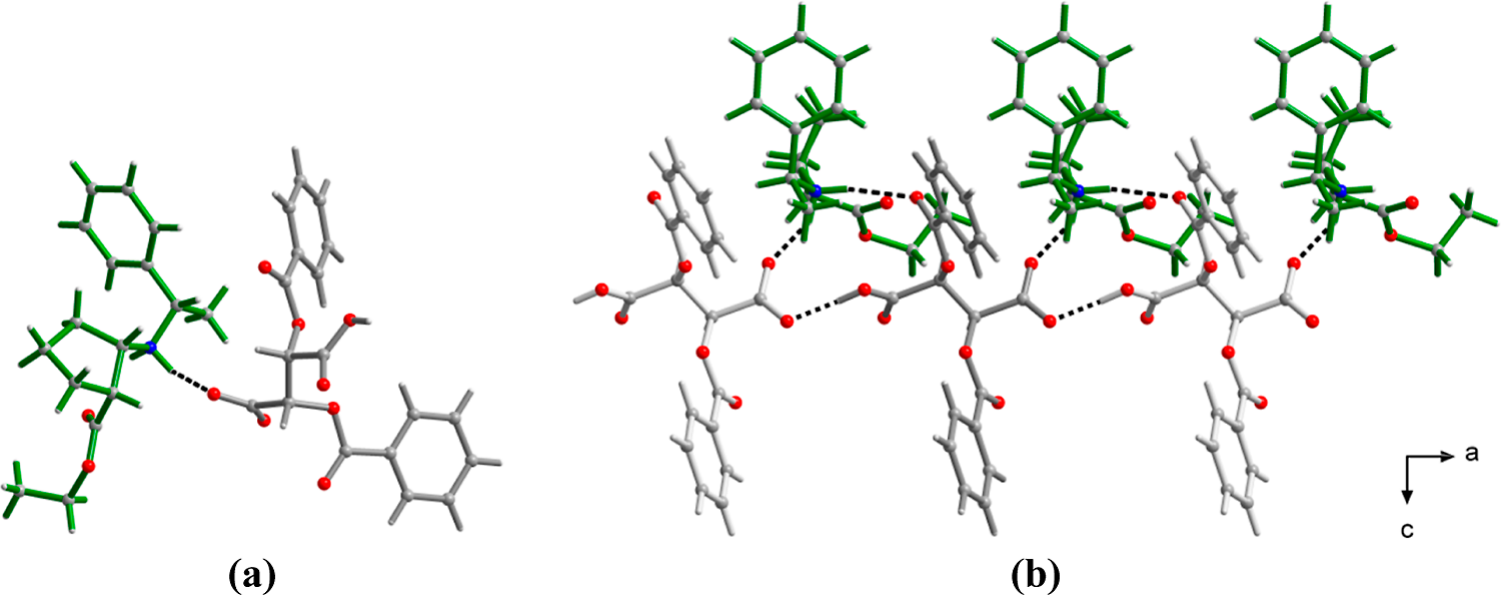
X-ray crystal structure of (*R*,*S*,*S*)-**2**•(D)-DBTA. Asymmetric unit (a) and hydrogen-bonded chain running down the crystallographic **a**-axis (b).

The stereochemistry of both isolated products (*S*,*S*,*S*)-**2**•HBr and (*R*,*S*,*S*)-**2**•(D)-DBTA was confirmed by single crystal X-ray diffraction (Figure 2 and S1). Furthermore, the crystal structure of the (*R*,*S*,*S*)-**2**•(D)-DBTA complex reveals strong interactions between two partners that include O–H···O, N–H···O, and C–H···O hydrogen bonds between the ester and amino groups of **2** and the carboxylate of DBTA, which explains the usefulness of DBTA for crystallization with product **2**.

The next challenge we had to overcome was the cleavage of the N–benzyl bond. According to the data from the literature, hydrogenolysis of such types of substrates may require high pressure,^35,36^ a large load of Pd catalyst,^37^ or can be accompanied by the formation of *N*-alkylated byproducts.^38^ We observed that hydrogenolysis of hydrobromide salt (*S*,*S*,*S*)-**2**•HBr proceeded smoothly in MeOH at 45 °C under the normal pressure of H_2_ in the presence of 10% w/w Pd on activated carbon (Scheme 1). Typically, complete conversion was achieved in 5 h. A similar rate of hydrogenolysis was observed for the salt (*R*,*S*,*S*)-**2**•(D)-DBTA. In this case, a small amount of methylated byproduct was identified in the NMR spectra when the reaction was run in methanol. However, the main complication we faced with this compound was a partial loss (as previously observed)^33^ of debenzylated *cis*-amino ester during its recovery from dibenzoyl tartaric salt. The best solution appeared to be a decomposition of the salt (*R*,*S*,*S*)-**2**•(D)-DBTA and isolation of free amine (*R*,*S*,*S*)-**2**, followed by its treatment with HBr and hydrogenolysis of the newly formed salt (*R*,*S*,*S*)-**2**•HBr under conditions identical to those of the *trans*-isomer (Scheme 1). Importantly, any traces of unreacted HBr have to be removed, as they affect the hydrogenolysis process.

Subsequently, ethyl esters of *cis* and *trans*-ACPC obtained in the form of hydrobromides were hydrolyzed under acidic conditions. Complete conversion to carboxylic acids was achieved by heating in hydrochloric acid (Scheme 1). According to NMR, carrying out the reaction below 80 °C did not cause the epimerization of the *trans*-isomer, while no signs of epimerization of the *cis*-isomer were noticed below 70 °C.

The amino acids obtained this way were converted to Fmoc derivatives **3** (Scheme 1), which are convenient for solid-phase peptide synthesis. *N*-(9-Fluorenylmethoxycarbonyloxy)succinimide, Fmoc-OSu, was used as a source of a protecting group. The reaction was performed in aqueous acetonitrile in the presence of KHCO_3_ as the base. According to our experiments, the use of potassium bicarbonate has an advantage over its sodium counterpart. It provided less viscous and more homogeneous mixtures during the reaction and easier extraction of the final products into the aqueous phase, probably due to the better solubility in water for the potassium salts of Fmoc-protected amino acids **3**. The total yield of Fmoc-protected *trans*-ACPC (*S*,*S*)-**3** is 34% for six subsequent steps, while the *cis* stereoisomer (*R*,*S*)-**3** was obtained with a 49% yield for five steps. Similarly, the opposite enantiomers of *cis* and *trans*-ACPC, the Fmoc derivatives (*S*,*R*)-**3** and (*R*,*R*)-**3**, were synthesized. (*R*)-α-phenylethylamine was used for the reductive amination of ketoester **1**, and crystallization with (−)-dibenzoyl-l-tartaric acid was applied to purify the intermediate amino ester (*S*,*R*,*R*)-**2**. It is worth underlining that the presented route to *cis* stereoisomers of ACPC may be easily and directly applied to both enantiomers, while published methods based on crystallization reveal weak points such as the lack of the opposite enantiomer when using dehydroabietylamine as a resolving base, possible regulations in the case of using ephedrine, and additional synthetic steps in the resolution with mandelic acid.^31−33^

**Figure 3:**
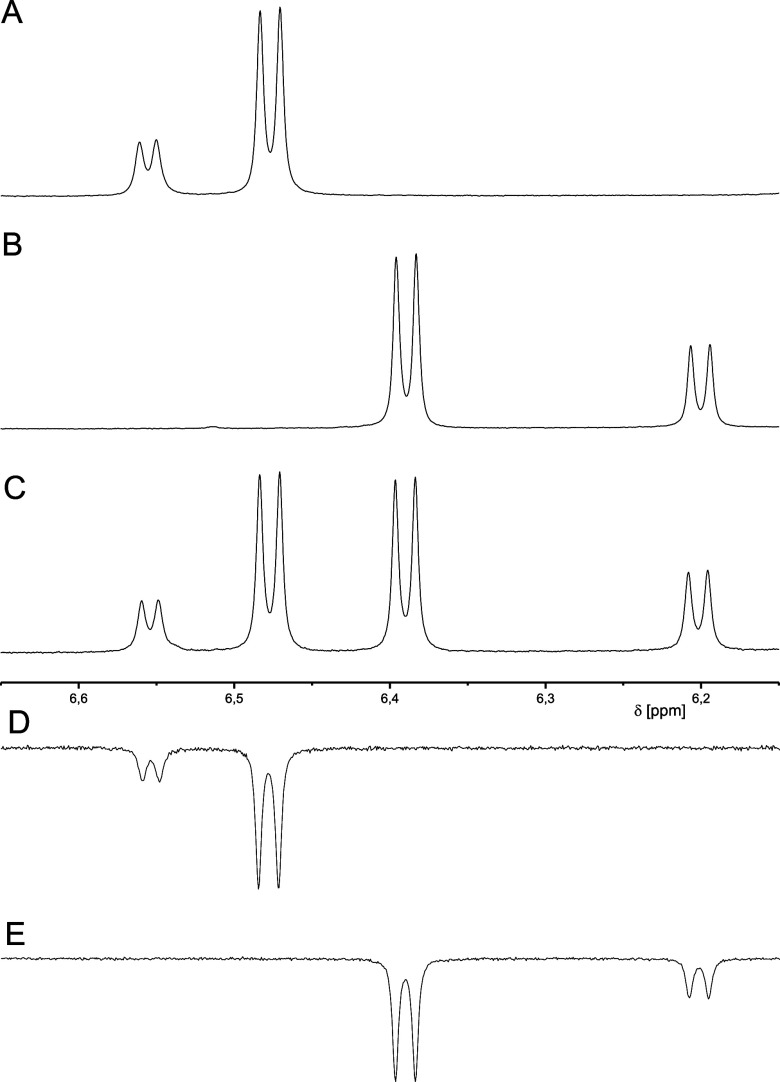
Fragments of ^1^H NMR spectra showing HN signals of (1*S*,2*R*)- (A), (1*R*,2*S*)- (B), and (*rac*)-Fmoc-*cis*-ACPC (C) in complex with quinine (2 eq.) in CDCl_3_ solution at 275 K. 1D NOE spectra of (*rac*)-Fmoc-*cis*-APCP after excitation of the major HN signal of the (1*S*,2*R*) enantiomer (D) or (1*R*,2*S*) enantiomer (E).

**Figure 4:**
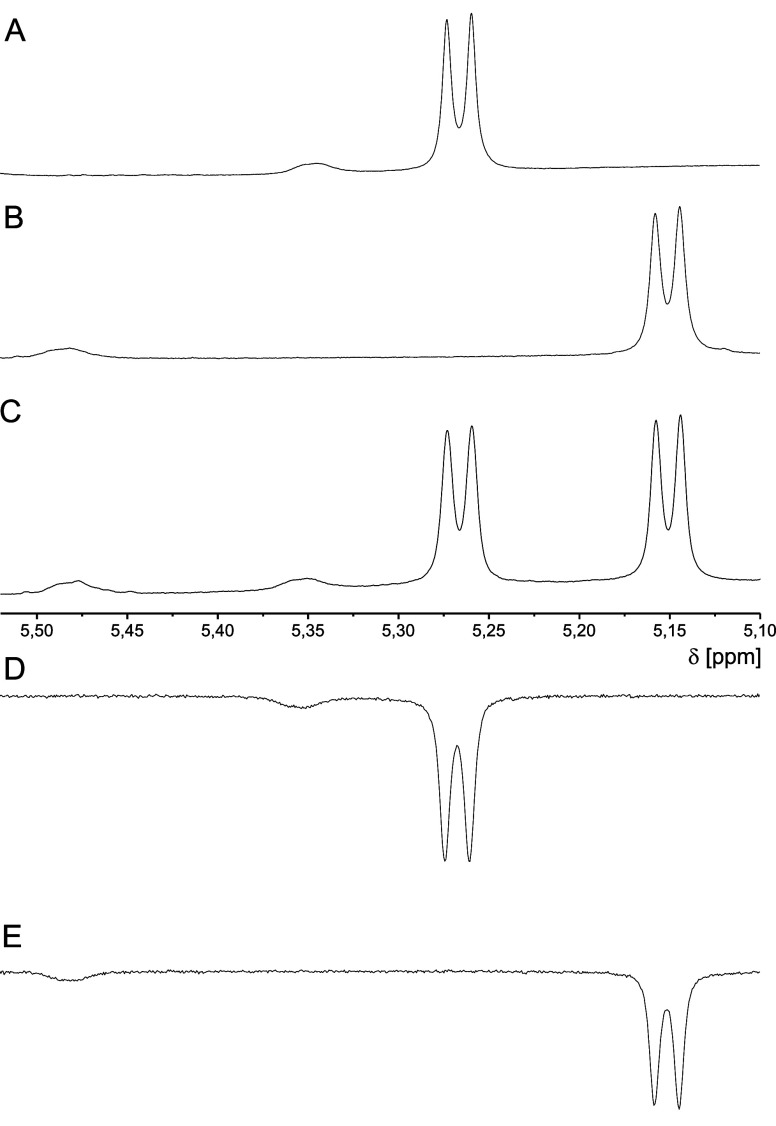
Fragments of ^1^H NMR spectra showing HN signals of (1*S*,2*S*)- (A), (1*R*,2*R*)- (B), and (*rac*)-Fmoc-*trans*-ACPC (C) in complex with quinine (2 equiv) in CDCl_3_ solution at 280 K. 1D NOE spectra of (*rac*)-Fmoc-*trans*-APCP after excitation of a major HN signal of the (1*S*,2*S*) enantiomer (D) or (1*R*,2*R*) enantiomer (E).

The enantiomeric purities of Fmoc derivatives that are used for peptide synthesis are crucial to obtaining the product successfully. Here, we propose to apply the CSA in ^1^H NMR measurements. The amino acid derivatives have been analyzed effectively using quinine, quinidine, or its derivatives.^39^ Analysis of ^1^H NMR spectra of Fmoc-*cis*-ACPC and Fmoc-*trans*-ACPC in CDCl_3_ indicated that both compounds are in the equilibrium of Fmoc-*cis*-carbamate and Fmoc-*trans*-carbamate isomers (Figure S3 and S4), which is visible by the presence of two resonances of the HN proton. In the case of Fmoc-*cis*-ACPC, the Fmoc-*cis*-carbamate isomer is more abundant (37%) than for Fmoc-*trans*-ACPC (approximately 10%). This feature of the studied analytes makes the analysis of the enantiomeric composition more difficult because CSA should discriminate between both entities present in the solution. Quinine (QN), quinidine (QD), and their *tert*-butyl carbamoyl derivatives (CQN and CQD) were tested as the CSA in this study. In all cases, the enantiodifferentiation of Fmoc-*cis*-ACPC and Fmoc-*trans*-ACPC was observed by doubling the resonances of the amide protons of the analytes. Still, in most cases, the observed picture did not allow for evaluating the enantiomeric composition reliably due to the overlapping of signals with each other or with those derived from the CSA (Figures S5 and S6). However, optimal conditions were found for both compounds after optimizing the excess of CSA and temperature (Figures 3 and 4). In the case of Fmoc-*cis*-ACPC, the addition of 2.0 equiv of QN and the measurement performed at 275 K allowed the baseline separation of signals derived from the (*S*,*R*)-**3** isomer (6.48 and 6.55 ppm) and (*R*,*S*)-**3** (6.20 and 6.39 ppm) (Figure 3). Unambiguous identification of signals deriving from each stereoisomer was achieved by comparison of spectra of individual enantiomers (Figure 3A,B) as well as selective excitation of major HN signals registering a 1D NOE spectrum (Figure 3D,E).

The enantiodiscrimination of Fmoc-*trans*-APCP was also effective using 2.0 equiv of QN at 280 K. The reliable evaluation of enantiomeric composition could be performed by the integration of sharp signals deriving from the major isomer at 5.27 ppm for (*S*,*S*)-**3** and 5.15 ppm for the (*R*,*R*)-**3** stereoisomer, as the amount of the minor isomer (peaks at 5.35 and 5.48 ppm) is the same for both stereoisomers (Figure 4).

## Conclusions

Herein, we present scalable syntheses of enantiopure *cis* and *trans*-2-aminocyclopentane carboxylic acids. The main features of our approach are access to all four stereoisomers of ACPC from the same precursor and through the same intermediate and avoidance of hazardous or expensive reagents, chromatographic separations, and high-pressure equipment. Moreover, a direct, reliable, and fast assessment of the enantiomeric purity using NMR spectroscopy was developed. Therefore, the presented synthetic and analytical methods for crucial building blocks for the synthesis of peptide foldamers will provide the possibility to significantly broaden the availability of this technology, in particular, to the community of medicinal chemists. Strategies such as foldamerization of conformationally labile peptides^16^ or the *de novo* design of biologically active peptides resistant to enzymatic degradation could become much more feasible.

## Experimental Section

### General Information

All solvents and reagents purchased from commercial suppliers (Sigma-Aldrich, Fluka, Merck, POCh, and Armar Chemicals) were of analytical grade and were used without further purification. 9-*O*-tert-Butylcarbamoylquinine and quinidine were synthesized according to standard procedures.^40^

NMR experiments were performed on a Jeol spectrometer operating at 400 MHz for ^1^H and 100 MHz for ^13^C (characterization of synthesized compounds) and on a Bruker Avance spectrometer operating at 600.58 MHz for ^1^H and 151.03 for ^13^C equipped with a 5 mm PA BBO probe (determination of enantiomeric purity). NMR spectra were recorded in CDCl_3_ (reference signals of the residual solvent at 7.26 ppm in ^1^H NMR), D_2_O (reference signals of the residual solvent at 4.79 ppm in ^1^H NMR), DMSO-*d*_6_ (reference signals of the residual solvent at 2.50 ppm in ^1^H NMR and 39.5 ppm in ^13^C NMR), and MeOH-*d*_4_ (reference signals of the residual solvent at 49.0 ppm in ^13^C NMR). Optical rotations were measured with a PolAAr 31. High-resolution mass spectra were recorded on a WATERS LCT Premier XE System with electrospray ionization and time-of-flight detection. TLC analysis was performed on Silica gel 60 F254 plates (Merck).

### Crude Ethyl [[(*S*)-1-Phenylethyl]amino]cyclopentanecarboxylate **2**

#### Step 1

Ethyl 2-oxocyclopentanecarboxylate **1** (50.0 g, 320.1 mmol) was dissolved in toluene (330 mL), and then isobutyric acid (32 mL, 352 mmol) and (*S*)-α-phenylethylamine (41.9 g, 345.7 mmol) were added subsequently. The mixture was heated in an oil bath at 70 °C for 2 h, and then the temperature was increased and a half amount of toluene was distilled together with the azeotropic removal of water. The residual amount of the mixture underwent reduction, as described below.

#### Step 2

In a separate flask equipped with a mechanical stirrer, NaBH4 (50 g, 1.32 mol) was added portion-wise to vigorously stirred ice-cooled isobutyric acid (600 mL). The internal temperature during the NaBH_4_ addition was controlled and did not exceed 10 °C. After this process was finished, the stirring was continued for an additional 30 min, and then the residue from step 1 was added using a dropping funnel. The reaction was continued in an ice bath, and the progress was monitored by TLC (eluent – CH_2_Cl_2_/MeOH, 25:1). If a low polar intermediate was detected after 3 h, then a new small portion of NaBH_4_ was added. When the reaction was complete, 200 mL of 5 M HCl was added slowly to the stirred ice-cooled mixture. As a result of the acidic workup, a white precipitate and a transparent pale yellow organic phase were formed. The organic layer was decanted, and the precipitate was washed with two 250 mL portions of diethyl ether. The combined organic phases were diluted with 1.5 L of hexane and extracted in a separatory funnel with 1 M HCl (5 × 300 mL). The combined aqueous layers were washed with hexane (100 mL), cooled in an ice bath, and basified to pH 10 by dropwise addition of 30% NaOH. The resulting mixture was extracted with diethyl ether (5 × 300 mL), and the extract was washed with brine. After drying over Na_2_SO_4_, the ether extract was evaporated in vacuum to give 80.0 g of the crude product **2**. A small amount of the product can be recovered additionally from the precipitate formed after the acidic workup of the reaction mixture. The solid material was dissolved in 2 L of 1 M HCl, and the solution was washed with hexane (100 mL). After the treatment with 30% NaOH and extraction as described above, up to 5 g of crude amino ester **2** was obtained. Total yield 85.0 g.

### Ethyl (1*S*,2*S*)-2-[[(*S*)-1-Phenylethyl]amino]cyclopentanecarboxylate Hydrobromide (*S*,*S*,*S*)-**2**•HBr

Sodium (17 g, 0.74 mol) was added to 800 mL of absolute ethanol, and the mixture was stirred vigorously until the reaction was complete (3–4 h). Then, crude amino ester **2** (∼170 g) prepared from 100 g (640.3 mmol) of ethyl 2-oxocyclopentanecarboxylate was added to sodium ethoxide solution, and the resulting mixture was stirred with heating at 30–35 °C (oil bath) for 18 h. After EtOH was removed in a vacuum at room temperature, the residue was cooled in an ice bath, and saturated NaHCO_3_ (750 mL) and brine (500 mL) were added. The product was extracted with Et_2_O (3 × 500 mL). After drying over Na_2_SO_4_ and being concentrated in a vacuum, the residue (approximately 160 g) was dissolved in Et_2_O (1 L), and 33% solution of HBr in acetic acid (121 mL, 0.67 mol) was added dropwise under cooling. A white precipitate was formed immediately. The mixture was left at rt for 2 h and then in the freezer overnight. After filtration, 170 g of the crude precipitate was obtained. This salt was recrystallized four times from acetonitrile (850–1000 mL of the solvent per crystallization; each time the mixture was left in the refrigerator for 12 h). The product was obtained as colorless needle crystals. The yield 78.0 g. Combined filtrates after crystallizations from acetonitrile were concentrated, and the residue was recrystallized two times from acetonitrile (200 mL) followed by two crystallizations from ethanol (50–60 mL). It additionally gave 10.0 g of pure material. Total yield of the salt (*S*,*S*,*S*)-**2**•HBr 88.0 g (40% from ethyl 2-oxocyclopentanecarboxylate). mp 216–219 °C; [α]_D_^25^ + 32 (*c* 1.0, MeOH); ^1^H NMR (400 MHz, D_2_O, δ): 7.41–7.53 (m, 5H; ArH), 4.47 (q, *J* = 6.8 Hz, 1H; PhC*H*), 4.06–4.19 (m, 2H; CH_3_C*H*_*2*_O), 3.77–3.83 (m, 1H; CH_2_C*H*NH), 3.03–3.08 (m, 1H; C*H*CO_2_Et), 2.03–2.19 (m, 2H; –[CH_2_]_3_–), 1.59–1.85 (m, 4H; –[CH_2_]_3_–), 1.66 (d, *J* = 6.8 Hz, 3H; C*H*_*3*_CH), and 1.23 (t, *J* = 7.2 Hz, 3H; C*H*_*3*_CH_2_O), NH proton not indicated; ^13^C{^1^H} NMR (100 MHz, D_2_O): δ 175.6, 135.8, 129.7, 129.5 (2C), 127.4 (2C), 62.6, 58.9, 57.6, 47.2, 30.6, 30.1, 23.8, 19.1, and 13.2; HRMS (ESI) *m*/*z*: [M + H]^+^ calcd for C_16_H_24_NO_2_, 262.1807; found, 262.1798.

### Ethyl (1*R*,2*S*)-2-[[(*S*)-1-Phenylethyl]amino]cyclopentanecarboxylate 2,3-Dibenzoyl-d-tartrate (*R*,*S*,*S*)-**2**•(D)-DBTA

Crude amino ester **2** (85 g) prepared from 50.0 g (320.1 mmol) of ethyl 2-oxocyclopentanecarboxylate was added dropwise to a hot solution of (2*S*,3*S*)-2,3-dibenzoyl-d-(+)-tartaric acid (118 g, 0.329 mol) in 1 L of acetonitrile. The mixture was constantly stirred using a magnetic stirrer and cooled to room temperature. A white precipitate formed, and the flask was placed in the refrigerator for 12 h. The precipitate was filtered and washed with cold acetonitrile. The collected solid material (approximately 150 g) was dissolved under heating in 80% aqueous acetonitrile (1 L), keeping the temperature during this process below the boiling point of the mixture. Hot water (1.3 L) was added to a transparent solution, and the mixture was cooled to room temperature. After the mixture was left to stand in the refrigerator for 12 h, the formed crystalline material was filtered. Crystallization from the mixture of acetonitrile–water was repeated to give the product as colorless needle crystals. The yield of (*R*,*S*,*S*)-**2**•(D)-DBTA 115.0 g (58% from ethyl 2-oxocyclopentanecarboxylate). mp 156–158 °C (with decomposition); [α]_D_^25^ + 34 (*c* 1.0, MeOH); ^1^H NMR (400 MHz, DMSO-*d*_6_): δ 7.93–8.02 (m, 4H; ArH), 7.66–7.70 (m, 2H; ArH), 7.53–7.57 (m, 4H; ArH), 7.40–7.44 (m, 2H; ArH), 7.34–7.38 (m, 2H; ArH), 7.27–7.31 (m, 1H; ArH), 5.76 (s, 2H; C*H*OBz); 4.12 (q, *J* = 7.1 Hz, 2H; CH_3_C*H*_*2*_O), 3.97–4.05 (m, 1H; PhC*H*), 3.08–3.14 (m, 1H; CH_2_C*H*NH), 2.93–2.97 (m, 1H; C*H*CO_2_Et), 1.30–1.87 (m, 6H; –[CH_2_]_3_–), 1.33 (d, *J* = 6.6 Hz, 3H; C*H*_*3*_CH), and 1.24 (t, *J* = 7.1 Hz, 3H; C*H*_*3*_CH_2_O), NH and CO_2_H protons not indicated; ^13^C{^1^H} NMR (100 MHz, MeOH-*d*_4_): δ 174.9, 171.4 (2C), 167.3 (2C), the signals 137.7, 134.4, 131.2, 131.0, 130.7, 130.5, 129.5, 128.9 belong to 18C_Ar_ overall, 75.0 (2C), 62.6, 59.8, 59.2, 45.0, 29.1 (2C), 22.1, 20.2, and 14.4. HRMS (ESI) *m*/*z*: [M + H]^+^ calcd for C_16_H_24_NO_2_, 262.1807; found, 262.1812; *m*/*z*: [M – H]^−^ calcd for C_18_H_13_O_8_, 357.0610; found, 357.0609.

### Fmoc-(1*S*,2*S*)-2-Aminocyclopentanecarboxylic Acid (1*S*,2*S*)-3

To a solution of salt (*S*,*S*,*S*)-**2**•HBr (60.0 g, 175.3 mmol) in MeOH (800 mL) under an argon atmosphere was added 10% palladium on activated carbon (6.0 g). The flask with the reaction mixture was evacuated and refilled with hydrogen (pressure 1.05 atm). The mixture was intensively stirred at 45 °C (oil bath temperature) for 5–6 h. Hydrogenolysis was complete when no more hydrogen consumption was observed, and according to TLC, (eluant – CH_2_Cl_2_/MeOH, 15:1), the UV-active starting material disappeared. After filtration through a pad of Celite, the solvent was removed in a vacuum. The residue was dissolved in 500 mL of 10% HCl and heated in an oil bath at 70 °C for 4 h. Then, the mixture was evaporated in a vacuum, and a new portion of 10% HCl was added. After heating in an oil bath at 60 °C for 12 h, the mixture was evaporated in a vacuum to dryness, and the solid residue was washed with ice-cooled acetone. It gives 34.4 g of (1*S*,2*S*)-2-aminocyclopentanecarboxylic acid in a salt form. ^1^H NMR (400 MHz, D_2_O): δ 3.88 (q, *J* = 7.4 Hz, 1H; C*H*NH_2_), 2.90–2.97 (m, 1H; C*H*CO_2_H), 2.13–2.23 (m, 2H; CH_2_), and 1.66–1.91 (m, 4H; CH_2_CH_2_), NH_2_ and CO_2_H protons not indicated; ^13^C{^1^H} NMR (100 MHz, D_2_O): δ 177.1, 53.9, 48.2, 30.4, 28.6, and 22.7. HRMS (ESI) *m*/*z*: [M + H]^+^ calcd for C_6_H_12_NO_2_, 130.0868; found, 130.0865.

This product was dissolved in water (400 mL), and then KHCO_3_ (68 g, 0.68 mol) was added portion-wise, followed by acetonitrile (350 mL) and Fmoc-OSu (57.3 g, 170 mmol). The reaction mixture was stirred at rt for 24 h. The mixture was diluted with water (2 L), and K_2_CO_3_ (approximately 10 g) was added. This resulted in a homogeneous solution, which was washed with EtOAc (2 × 200 mL). Then, the organic phase was extracted with a 5% solution of K_2_CO_3_ (100 mL), and this extraction was combined with the main water phase. Further, the water phase was acidified with 1 M HCl, and the precipitated product was extracted with EtOAc (3 × 500 mL). Organic extracts were washed with diluted HCl (3 × 1 L) and brine (200 mL), dried over Na_2_SO_4_, and concentrated in a vacuum. The residue was dissolved in an aqueous solution (1.5 L) containing 50 g (0.5 mol) of KHCO_3_ and 15 g (0.11 mol) of K_2_CO_3_. The mixture was filtered from the traces of insoluble material and added dropwise to stirred 1 M HCl (1 L). A white precipitate formed, and the mixture was left in the refrigerator overnight. Precipitate was filtered and washed with diluted HCl and distilled water. After air drying, the residual water was removed by lyophilization. The product was obtained as a white powder. The yield 53.0 g (86%). mp 160–172 °C (with decomposition); [α]_D_^25^ + 36 (*c* 1.0, MeOH). The value of optical rotation and NMR spectra were in agreement with the previous report.^31^

### Fmoc-(1*R*,2*S*)-2-Aminocyclopentanecarboxylic Acid (1*R*,2*S*)-**3**

The salt (*R*,*S*,*S*)-**2**•(D)-DBTA (115 g, 185.6 mmol) was treated with diethyl ether (500 mL) and 1L of aqueous solution containing KHCO_3_ (50 g) and K_2_CO_3_ (50 g). A two-phase mixture was stirred until all of the solids were dissolved. The organic phase was separated, and the aqueous phase was extracted with diethyl ether (3 × 150 mL). Combined extracts were washed with a 10% solution of K_2_CO_3_ and with brine, dried over Na_2_SO_4_, and evaporated in a vacuum to give free amine (*R*,*S*,*S*)-**2** as a colorless liquid (48.5 g). ^1^H NMR (400 MHz, 0.1 M HCl in D_2_O): δ 7.41–7.46 (m, 5H; ArH), 4.41 (q, *J* = 6.8 Hz, 1H; PhC*H*), 4.10–4.22 (m, 2H; CH_3_C*H*_*2*_O), 3.40–3.46 (m, 1H; CH_2_C*H*NH), 3.11–3.16 (m, 1H; C*H*CO_2_Et), 1.45–1.97 (m, 6H; -[CH_2_]_3_-), 1.62 (d, *J* = 6.8 Hz, 3H; C*H*_*3*_CH), and 1.21 (t, *J* = 7.2 Hz, 3H; C*H*_*3*_CH_2_O), NH proton not indicated; ^13^C{^1^H} NMR (100 MHz, D_2_O): δ 174.9, 135.6, 129.8, 129.5 (2C), 127.6 (2C), 62.5, 58.3, 57.4, 43.6, 27.8, 27.7, 20.7, 18.6, and 13.2.

Free amine was dissolved in EtOAc (500 mL), and a solution of HBr in AcOH (33% by weight, 40 mL, approximately 220 mmol) was added. The mixture was evaporated in a vacuum to dryness to give the hydrobromide salt of amine (*R*,*S*,*S*)-**2** as a viscous oil. The residue was coevaporated with a fresh portion of EtOAc to remove unreacted HBr. This hydrobromide underwent hydrogenolysis followed by ester hydrolysis as described for the *trans*-isomer to give 35.0 g of (1*R*,2*S*)-2-aminocyclopentanecarboxylic acid in a salt form. ^1^H NMR (400 MHz, D_2_O): δ 3.82–3.86 (m, 1H; C*H*NH_2_), 3.10–3.16 (m, 1H; C*H*CO_2_H), 2.08–2.20 (m, 2H; CH_2_), and 1.69–1.99 (m, 4H; CH_2_CH_2_), NH_2_ and CO_2_H protons not indicated; ^13^C{^1^H} NMR (100 MHz, D_2_O): δ 176.6, 52.7, 45.5, 29.7, 27.2, and 21.2. HRMS (ESI) *m*/*z*: [M + H]^+^ calcd for C_6_H_12_NO_2_, 130.0868; found, 130.0865.

This intermediate was further converted into Fmoc-protected (1*R*,2*S*)-2-aminocyclopentanecarboxylic acid (1*R*,2*S*)-**3** as described above for the *trans*-isomer (1*S*,2*S*)-**3**. The yield 55.5 g (85%). White powder, mp 134–137 °C; [α]_D_^25^ −31 (*c* 1.0, CHCl_3_). NMR spectra^41^ and the value of optical rotation^42^ were in agreement with previous reports.

### Fmoc-(1*R*,2*R*)-**2**-Aminocyclopentanecarboxylic Acid (1*R*,2*R*)-**3** and Fmoc-(1*S*,2*R*)-**2**-Aminocyclopentanecarboxylic Acid (1*S*,2*R*)-**3**

Two other stereoisomers, (1*R*,2*R*)-**3** and (1*S*,2*R*)-**3**, were synthesized in the same manner as their enantiomers, using ethyl 2-oxocyclopentanecarboxylate **1** and (*R*)-α-phenylethylamine as starting materials. (2*R*,3*R*)-2,3-Dibenzoyl-l-(−)-tartaric acid was utilized to isolate the corresponding stereopure intermediate for *cis*-ACPC (*S*,*R*,*R*)-**2**. Spectroscopic characteristics were identical to those of compounds (1*S*,2*S*)-**3** and (1*S*,2*R*)-**3**.

### NMR Measurements

The measurements were performed at different temperatures: 298, 285, 280, and 275 K. The temperature was controlled to 0.1 K. Standard 1D NOESY spectra were recorded using selective refocusing with a shaped pulse: mixing time—0.6 s, relaxation delay—2 s, and number of scans—256. All measurements were made in CDCl_3_ solution containing TMS as a standard. Samples for analysis were prepared by diluting appropriate proportions of 100 mM stock solutions of individual Fmoc-2-aminocyclopentanecarboxylic acid stereoisomers and the chiral selector (quinine, QN, quinidine, QD, and their 9-*O*-tert-butylcarbamoyl derivatives). The final concentration of 10 mM was used for Fmoc-ACPC and 10 or 20 mM for CSA.

## Data Availability

The data underlying this study are available in the published article and its Supporting Information.

## Supporting Information Available

Experimental details of X-ray crystal structures measurement and solving, details of crystal structures of compounds (*R*,*S*,*S*)-**2**•(D)-DBTA and (*S*,*S*,*S*)-**2**•HBr, figures with crystal structures, and copies of ^1^H and ^13^C NMR spectra for compounds (*R*,*S*,*S*)-**2**•(D)-DBTA, (*R*,*S*,*S*)-**2** (in the form of HCl salt), (*S*,*S*,*S*)-**2**•HBr, (1*S*,2*S*)-ACPC (in the form of hydrogen halide salt), and (1*S*,2*S*)-**3**, (1*R*,2*S*)-ACPC (in the form of hydrogen halide salt), (1*R*,2*S*)-**3** (PDF)
