# Supplementary material for: Scalable Synthesis of All Stereoisomers of 2-Aminocyclopentanecarboxylic Acid—A Toolbox for Peptide Foldamer Chemistry
Source: J Org Chem. 2024 Mar 28;89(7):4760–7. doi: 10.1021/acs.joc.3c02991 (PMC11002926; doi:10.1021/acs.joc.3c02991)
Supplement: Supplementary file 1 — jo3c02991_si_001.pdf [file jo3c02991_si_001.pdf]

Supporting Information

for

Scalable synthesis of all stereoisomers of 2-aminocyclopentanecarboxylic acids – a toolbox for peptide foldamer chemistry

Vitaly Kovalenko[a], Ewa Rudzińska-Szostak[a], Katarzyna Ślepokura[b], Łukasz Berlicki*[a]

[a]Department of Bioorganic Chemistry, Wrocław University of Science and Technology, Wyb. Wyspiańskiego 27, 50-370 Wrocław, Poland

[b]University of Wrocław, Faculty of Chemistry, 14 F. Joliot-Curie, 50-383 Wrocław, Poland

* Corresponding author: lukasz.berlicki@pwr.edu.pl

Table of Contents

X-ray crystallography S3
Table S1. Crystal data for (*S*,*S*,*S*)-**2**•HBr and (*R*,*S*,*S*)-**2**•(D)-DBTA S4
Table S2. Hydrogen-bond geometry (Å, °) for (*S*,*S*,*S*)-**2**•HBr S5
Table S3. Hydrogen-bond geometry (Å, °) for (*R*,*S*,*S*)-**2**•(D)-DBTA S5
Figure S1. Asymmetric units of crystals (*S*,*S*,*S*)-**2**•HBr and (*R*,*S*,*S*)-**2**•(D)-DBTA S6
Figure S2. Arrangement of the ions in crystals (*S*,*S*,*S*)-**2**•HBr and (*R*,*S*,*S*)-**2**•(D)-DBTA S7
Figure S3. Fragment of ^{1}H NMR spectrum of Fmoc-*cis*-ACPC with HN protons signals (with integrals) of Fmoc-*trans*-carbamate and Fmoc-*cis*-carbamate stereoisomers at 280K. S8
Figure S4. Fragment of ^{1}H NMR spectrum of Fmoc-*trans*-ACPC with HN proton signal of Fmoc-*trans*-carbamate and Fmoc-*cis*-carbamate stereoisomers at 280K. S8
Figure S5. Fragments of ^{1}H NMR spectra showing HN signals of a mixture of (1S,2R)- (•) and (1R,2S)-Fmoc-*cis*-ACPC (♦) in complex with 1 eq. of quinine (A), quinidine (B), *tert*-butyl carbamoylquinine (C) and *tert*-butyl carbamoylquinidine (D) in $CDCl_3$ solution at 285K. S9
Figure S6. Fragments of ^{1}H NMR spectra showing HN signals of a mixture of (1S,2S)- (◊) and (1R,2R)-Fmoc-*trans*-ACPC ($_{o}$) in complex with 1 eq. of quinine (A), quinidine (B), *tert*-butyl carbamoylquinine (C) and *tert*-butyl carbamoylquinidine (D) in $CDCl_3$ solution at 285K. S10
^{1}H/^{13}C NMR of (*R*,*S*,*S*)-**2**•(D)-DBTA S11
^{1}H/^{13}C NMR of (*R*,*S*,*S*)-**2** (in the form of HCl salt) S12
^{1}H/^{13}C NMR of salt (*S*,*S*,*S*)-**2**•HBr S13
^{1}H/^{13}C NMR of (1*S*,2*S*)-ACPC (in the form of hydrogen halide salt) S14
^{1}H/^{13}C NMR of (*S*,*S*)-**3** S15
^{1}H/^{13}C NMR of (1*R*,2*S*)-ACPC (in the form of hydrogen halide salt) S16
^{1}H/^{13}C NMR of (*R*,*S*)-**3** S17
References S18

X-ray crystallography

X-ray quality crystals of (*S*,*S*,*S*)-**2**•HBr and (*R*,*S*,*S*)-**2**•(D)-DBTA were grown by slow isothermal evaporation from saturated solution in acetonitrile, or acetonitrile/water (8:15, v/v, ratio), respectively.

Diffraction data for the crystals were collected at 100 or 150 K on a κ-geometry Rigaku XtaLAB Synergy DW diffractometer with Cu *K*α radiation (ω scans, rotating-anode X-ray source). Diffraction pattern of the (*S*,*S*,*S*)-**2**•HBr crystal at 100 K was of very poor quality. Variable-temperature diffraction measurements suggested a phase transition at about 130-140 K. Therefore a crystal structure determined at 150 K is presented here. Data collections, cell refinements, data reductions and analyses for the crystals, including numerical and/or empirical (multi-scan) absorption corrections, were carried out with *CrysAlisPRO*.[1] With the use of Olex2,[2] structures were solved with *SHELXT* program[3] employing dual-space algorithm, and refined on F^2 by a full-matrix least-squares procedure using *SHELXL* program[4] with anisotropic displacement parameters for the ordered (fully occupied) and more occupied (site occupation factor, SOF > 0.5) positions of disordered non-H atoms. The absolute structure of the crystals was determined based on the known absolute configuration of the compounds, and confirmed by anomalous-dispersion effects in diffraction measurements and the Flack parameters.[5]

Cyclopentane ring and ethoxy group of the organic cation in (*S*,*S*,*S*)-**2**•HBr were found to be partially disordered and were refined in two positions each, with SOFs = 0.765(17)/0.235(17) for the ring and 0.707(14)/0.293(14) for the CH_3–CH_2– fragment. H atoms in the crystal structures of (*S*,*S*,*S*)-**2**•HBr and (*R*,*S*,*S*)-**2**•(D)-DBTA were found in the difference Fourier maps or were included using geometrical considerations. In the final refinement cycles, all C-bound, O-bound and most of the N-bound H atoms were repositioned in their calculated positions and were refined using a riding model, with C–H = 0.95-1.00 Å, O–H = 0.84 Å and N–H = 0.91 Å, and with $U_{iso}(H)$ = $1.2U_{eq}(N,C)$ for NH_2, CH, CH_2 or $1.5U_{eq}(O,C)$ for OH and CH_3. H atoms from NH_2 group in (*R*,*S*,*S*)-**2**•(D)-DBTA were refined freely.

Crystals of (*S*,*S*,*S*)-**2**•HBr are isomorphous with the chloride analogue deposited at the Cambridge Crystallographic Data Centre as a *CSD Private Communication* (Refcode BAJSIS).[6] The position of the organic cation in the unit cell is the same in both structures.

Figures presenting molecular structures were made using the *DIAMOND* program.[7] Details of structures refinements are given in Table S1 and the crystallographic information files (CIFs)

deposited at the Cambridge Crystallographic Data Centre (CCDC Nos. 2287356 and 2287357) and provided as Supplementary Information.

Table S1. Crystal data for (*S*,*S*,*S*)-**2**•HBr and (*R*,*S*,*S*)-**2**•(D)-DBTA

	(*S*,*S*,*S*)-**2**•HBr	(*R*,*S*,*S*)-**2**•(D)-DBTA
CCDC No.	2287356	2287357
Chemical formula	$(C_{16}H_{24}NO_2)Br$	$(C_{16}H_{24}NO_2)(C_{18}H_{13}O_8)$
M_r	342.27	619.64
Crystal system, space group	Monoclinic, *C*2	Orthorhombic, $P2_12_12_1$
Temperature (K)	150	100
a, *b*, *c* (Å)	16.035(3), 7.6583(11), 14.743(2)	7.7895(11), 16.928(3), 24.097(4)
α, β, γ (°)	90, 108.44(2), 90	90, 90, 90
V (Å^3)	1717.5(5)	3177.4(9)
Z	4	4
Radiation type	Cu *K*α	Cu *K*α
μ (mm^{-1})	3.27	0.79
Crystal size (mm)	0.26 × 0.06 × 0.02	0.14 × 0.11 × 0.08
Diffractometer	Rigaku XtaLAB Synergy R, DW system, with HyPix-Arc 150 detector	Rigaku XtaLAB Synergy R, DW system, with HyPix-Arc 150 detector
Absorption correction	Gaussian	Multi-scan
T_{min}, T_{max}	0.383, 1.000	0.839, 1.000
No. of measured, independent and observed [$I > 2\sigma(I)$] reflections	10825, 3306, 3116	24591, 6176, 5972
R_{int}	0.029	0.024
$(\sin \theta/\lambda)_{max}$ (Å^{-1})	0.621	0.622
$R[F^2 > 2\sigma(F^2)]$, $wR(F^2)$, S	0.031, 0.084, 1.02	0.026, 0.064, 1.04
No. of reflections	3306	6176
No. of parameters	198	418
No. of restraints	10	0
H-atom treatment	H-atom parameters constrained	H atoms treated by a mixture of independent and constrained refinement
$\Delta\rho_{max}$, $\Delta\rho_{min}$ (e Å^{-3})	0.41, −0.39	0.21, −0.16
Absolute structure parameter	−0.027(17)	−0.02(5)

Computer programs: *CrysAlis PRO* 1.171.42.72a (Rigaku OD, 2022), SHELXT-2014/5 (Sheldrick, 2015), *SHELXL2014*/7 (Sheldrick, 2015).

Table S2. Hydrogen-bond geometry (Å, º) for (*S*,*S*,*S*)-**2**•HBr

D—H···*A*	*D*—H	H···*A*	*D*···*A*	*D*—H···*A*
N11—H11*A*···Br1	0.91	2.35	3.260(3)	173
N11—H11*B*···Br1^{i}	0.91	2.36	3.263(3)	175
C12—H12···Br1^{ii}	1.00	2.76	3.751(5)	170

Symmetry codes: (i) −*x*+1/2, *y*−1/2, −*z*+1; (ii) *x*, *y*−1, *z*.

Table S3. Hydrogen-bond geometry (Å, º) for (*R*,*S*,*S*)-**2**•(D)-DBTA

D—H···*A*	*D*—H	H···*A*	*D*···*A*	*D*—H···*A*
O6*A*—H6*A*···O2*A*^{i}	0.84	1.66	2.4866(16)	166
N11—H11*A*···O8*A*^{ii}	0.95(2)	2.34(2)	3.2275(19)	155.4(18)
N11—H11*B*···O1*A*	0.94(2)	1.78(2)	2.6315(18)	149(2)
C9—H9*A*···O1*A*^{iii}	0.99	2.57	3.483(2)	153
C10—H10*C*···O5*A*^{iv}	0.98	2.44	3.283(2)	144
C15*A*—H15*A*···O6*A*^{v}	0.95	2.54	3.243(2)	131
C16—H16···O4*A*^{vi}	0.95	2.45	3.335(2)	156
C18—H18···O4*A*^{vii}	0.95	2.45	3.348(2)	157

Symmetry codes: (i) *x*−1, *y*, *z*; (ii) *x*+1, *y*, *z*; (iii) *x*+1/2, −*y*+1/2, −*z*+1; (iv) *x*+3/2, −*y*+1/2, −*z*+1; (v) *x*+1/2, −*y*+3/2, −*z*+1; (vi) −*x*+3/2, −*y*+1, *z*−1/2; (vii) −*x*+1/2, −*y*+1, *z*−1/2.

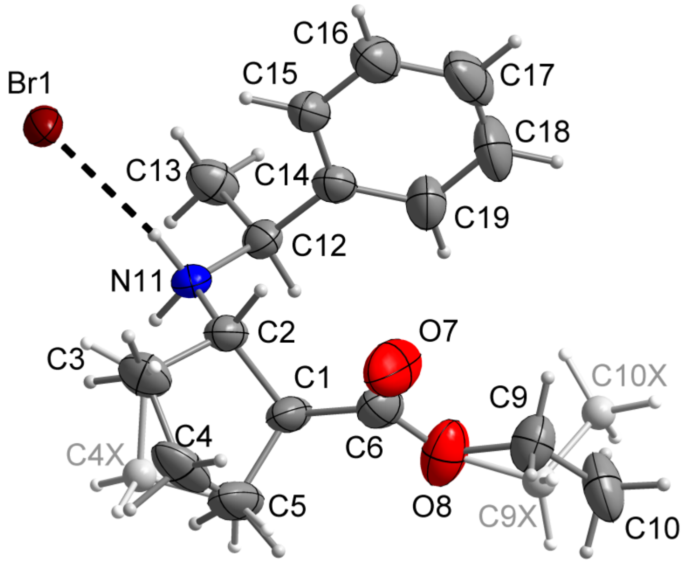

(a)

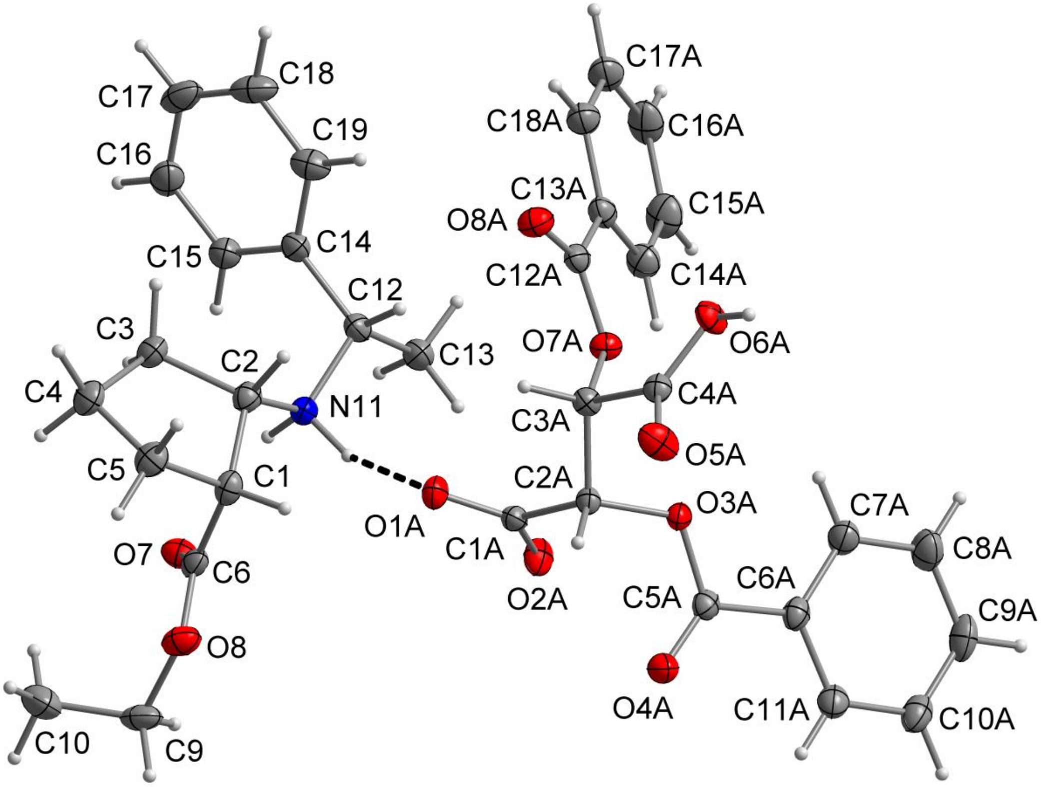

(b)

Figure S1. Asymmetric units of crystals (*S*,*S*,*S*)-**2**•HBr (a) and (*R*,*S*,*S*)-**2**•(D)-DBTA (b) showing the atom-numbering schemes and the symmetry-independent hydrogen bonds (dashed lines). Displacement ellipsoids are shown at the 50% probability level. The positions of disordered fragments in (*S*,*S*,*S*)-**2**•HBr are shown in different shades of grey.

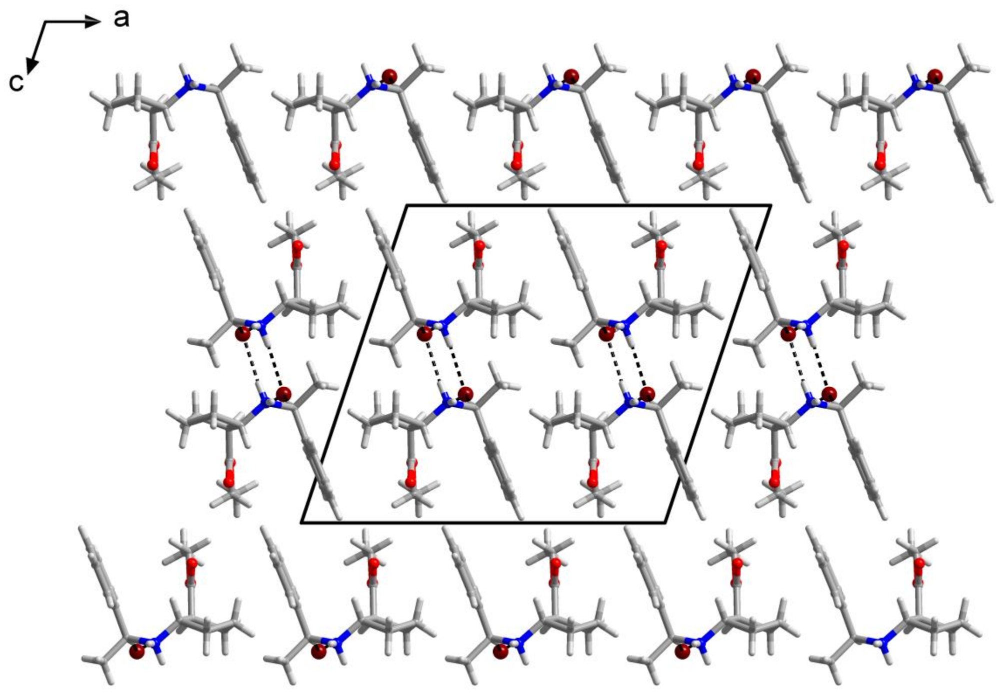

(a)

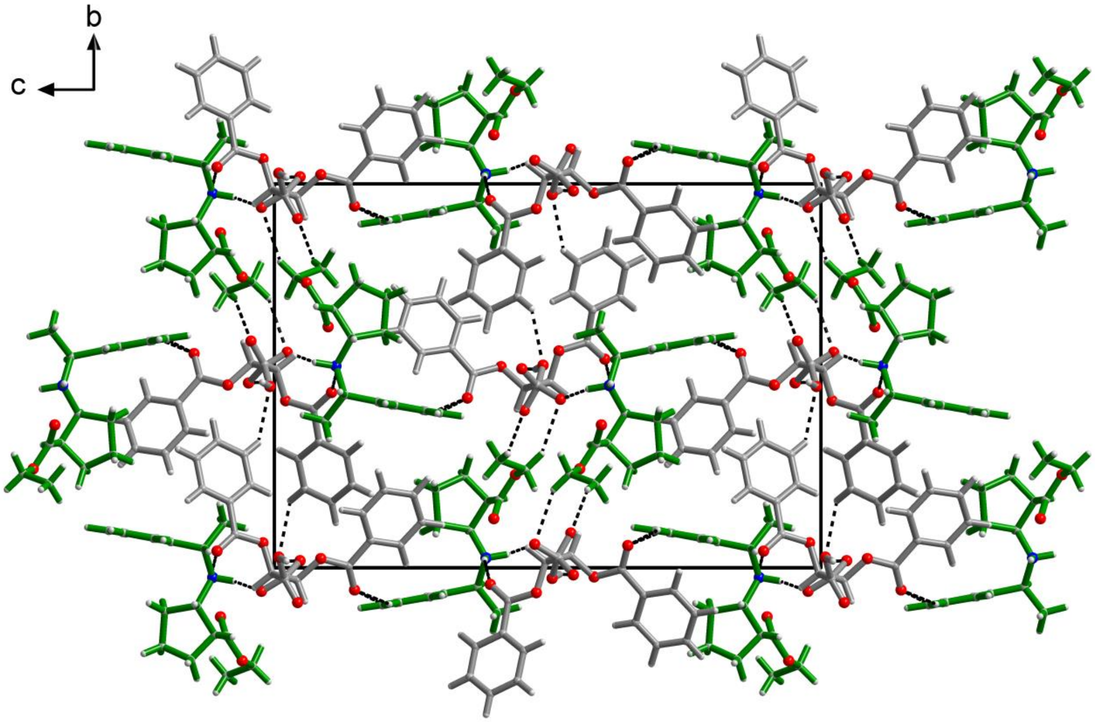

(b)

Figure S2. Arrangement of the ions in crystals (*S*,*S*,*S*)-**2**•HBr (a) and (*R*,*S*,*S*)-**2**•(D)-DBTA (b). Hydrogen bonds are shown with dashed lines. Disorder in (*S*,*S*,*S*)-**2**•HBr is omitted for clarity.

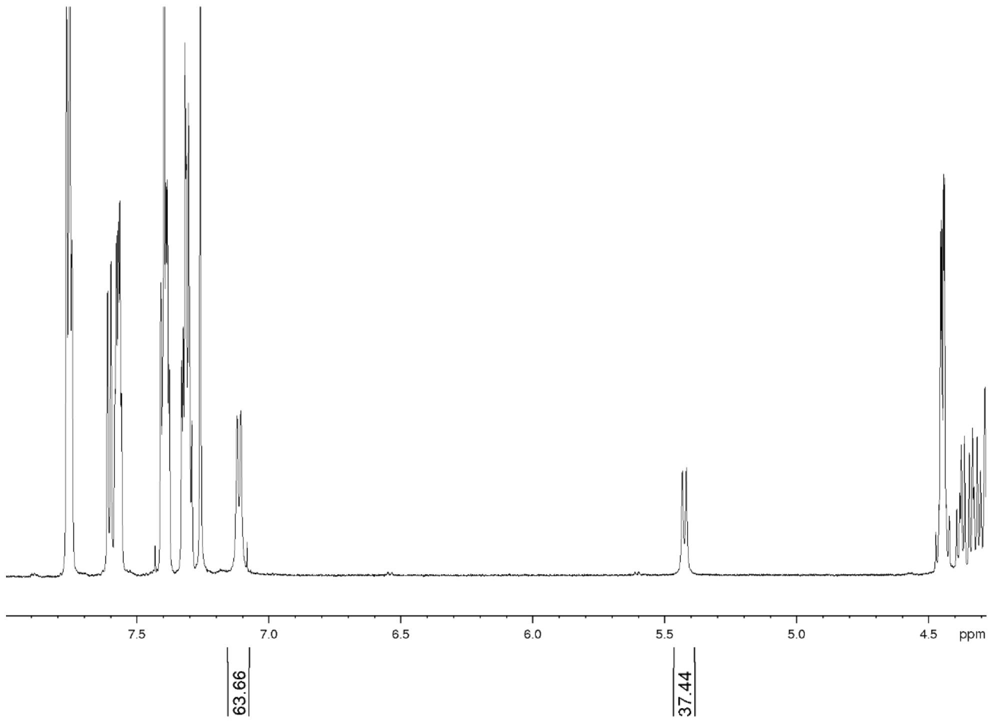

Figure S3. Fragment of ^{1}H NMR (600MHz) spectrum of Fmoc-*cis*-ACPC with HN protons signals (with integrals) of Fmoc-*trans*-carbamate and Fmoc-*cis*-carbamate stereoisomers at 280K.

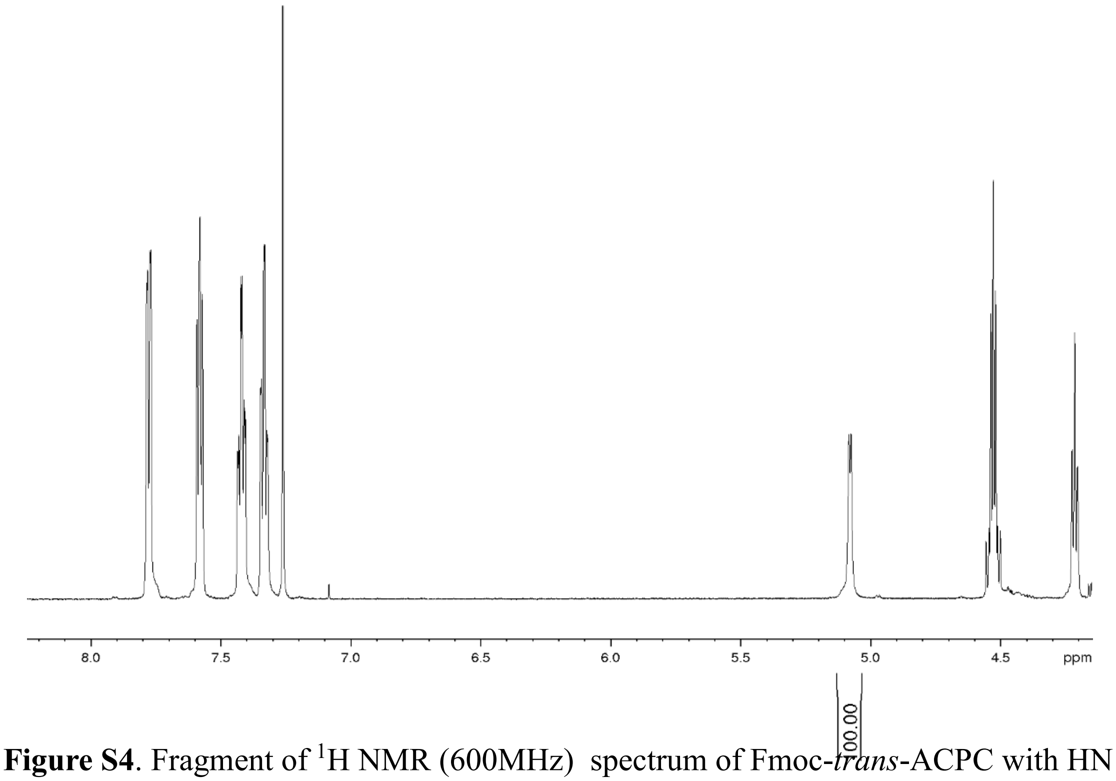

Figure S4. Fragment of ^{1}H NMR (600MHz) spectrum of Fmoc-*trans*-ACPC with HN proton signal of Fmoc-*trans*-carbamate and Fmoc-*cis*-carbamate stereoisomers at 280K.

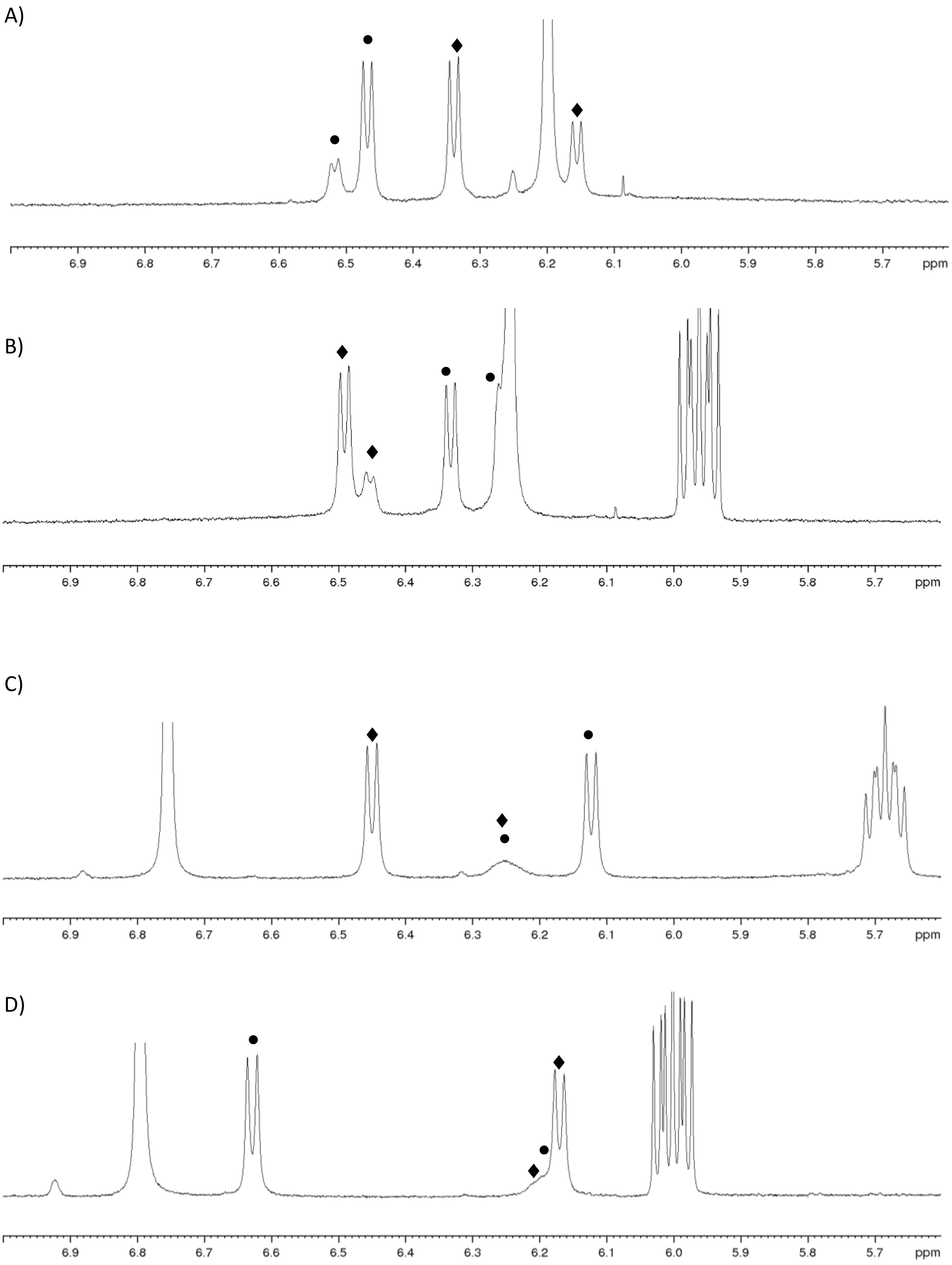

Figure S5. Fragments of ^{1}H NMR (600MHz) spectra showing HN signals of a mixture of (1S,2R)-(•) and (1R,2S)-Fmoc-*cis*-ACPC (♦) in complex with 1 eq. of quinine (A), quinidine (B), *tert*-butyl carbamoylquinine (C) and *tert*-butyl carbamoylquinidine (D) in $CDCl_3$ solution at 285K.

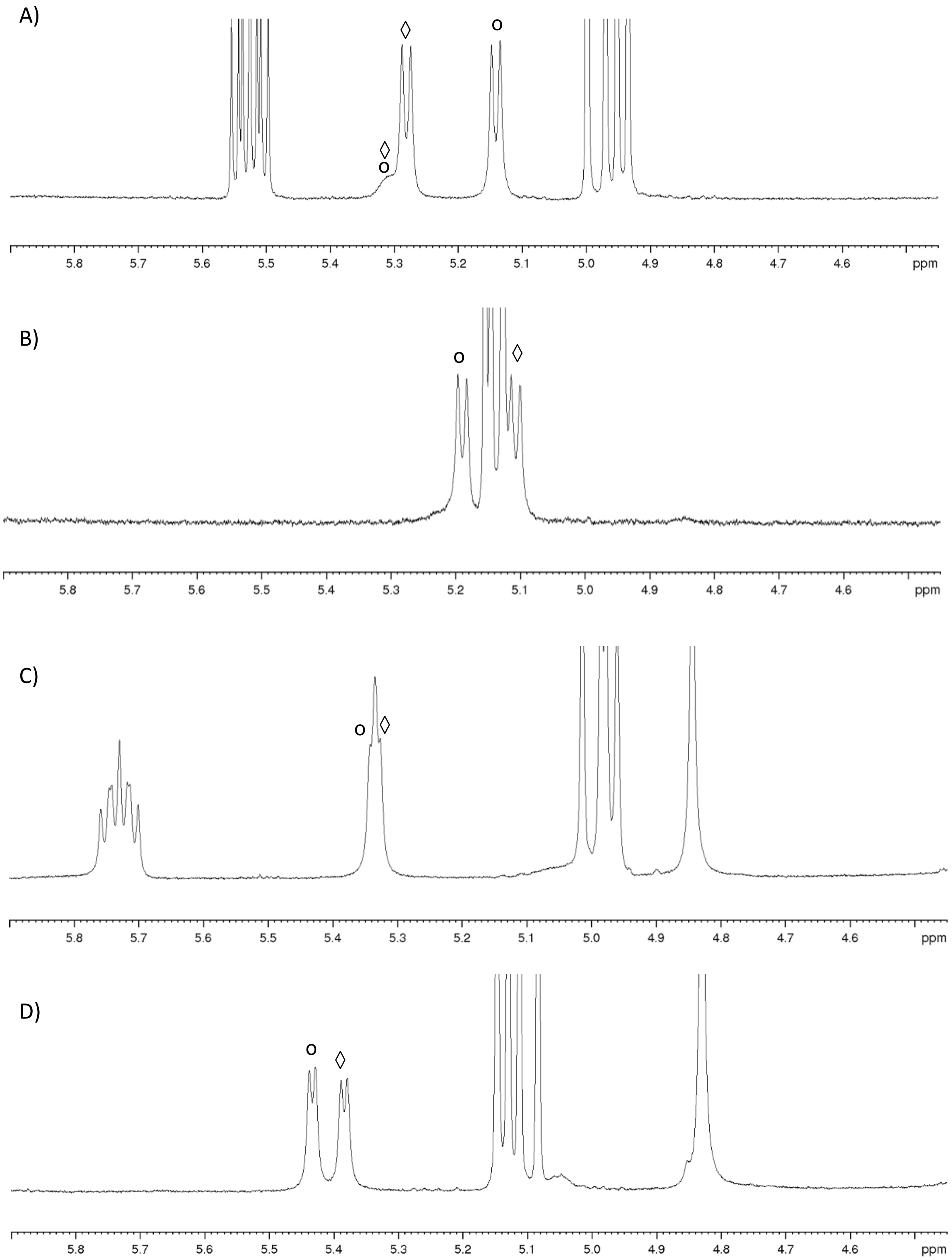

Figure S6. Fragments of ^{1}H NMR (600MHz) spectra showing HN signals of a mixture of (1S,2S)-(◊) and (1R,2R)-Fmoc-*trans*-ACPC ($_\mathrm{o}$) in complex with 1 eq. of quinine (A), quinidine (B), *tert*-butyl carbamoylquinine (C) and *tert*-butyl carbamoylquinidine (D) in $CDCl_3$ solution at 285K.

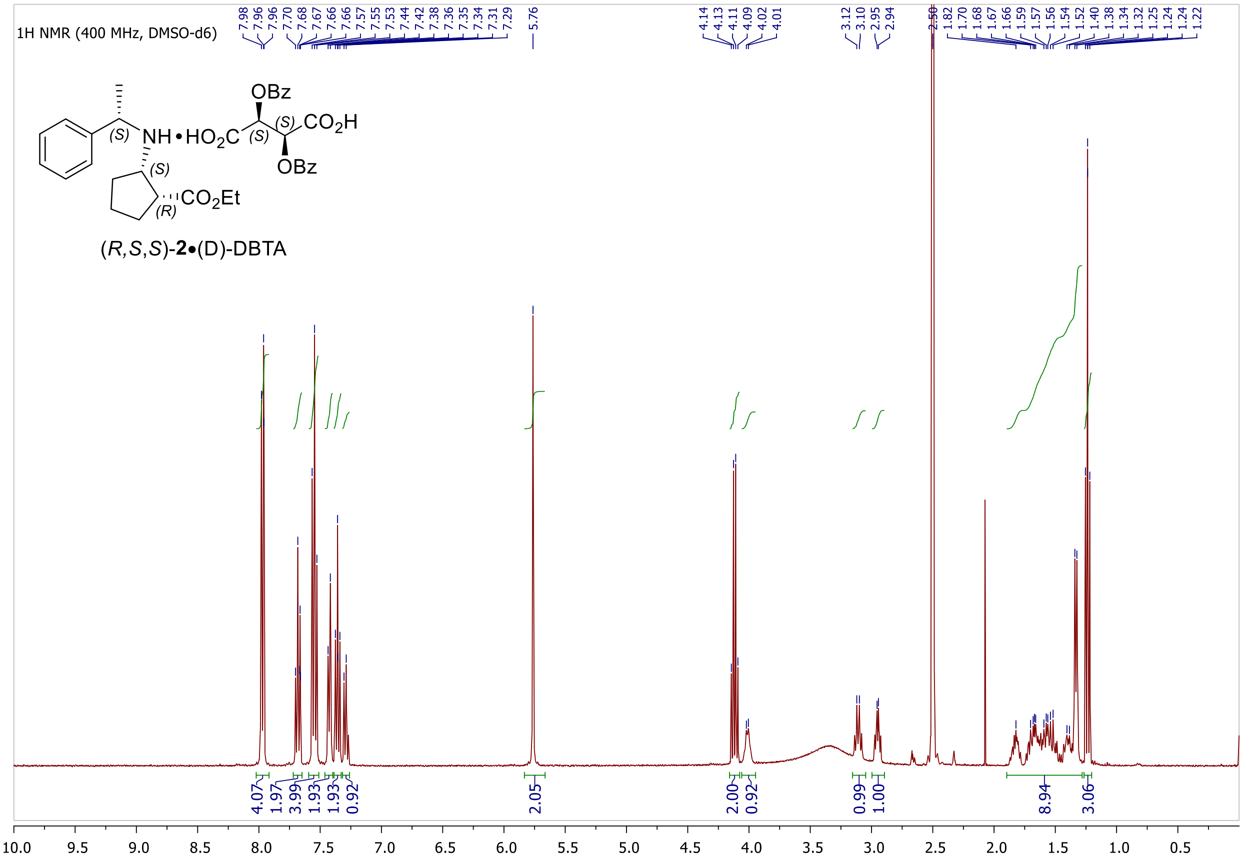
1H NMR (400 MHz, DMSO-d6)
(R,S,S)-2•(D)-DBTA
7.98 7.96 7.96 7.70 7.68 7.67 7.66 7.66 7.57 7.55 7.53 7.44 7.42 7.38 7.36 7.35 7.34 7.31 7.29
5.76
4.14 4.13 4.11 4.09 4.02 4.01
3.12 3.10 2.95 2.94
2.50
1.82 1.70 1.68 1.67 1.66 1.59 1.57 1.56 1.54 1.52 1.40 1.38 1.34 1.32 1.25 1.24 1.24 1.22
4.07 1.97 3.99 1.93 1.93 0.92 2.05 2.00 0.92 0.99 1.00 8.94 3.06

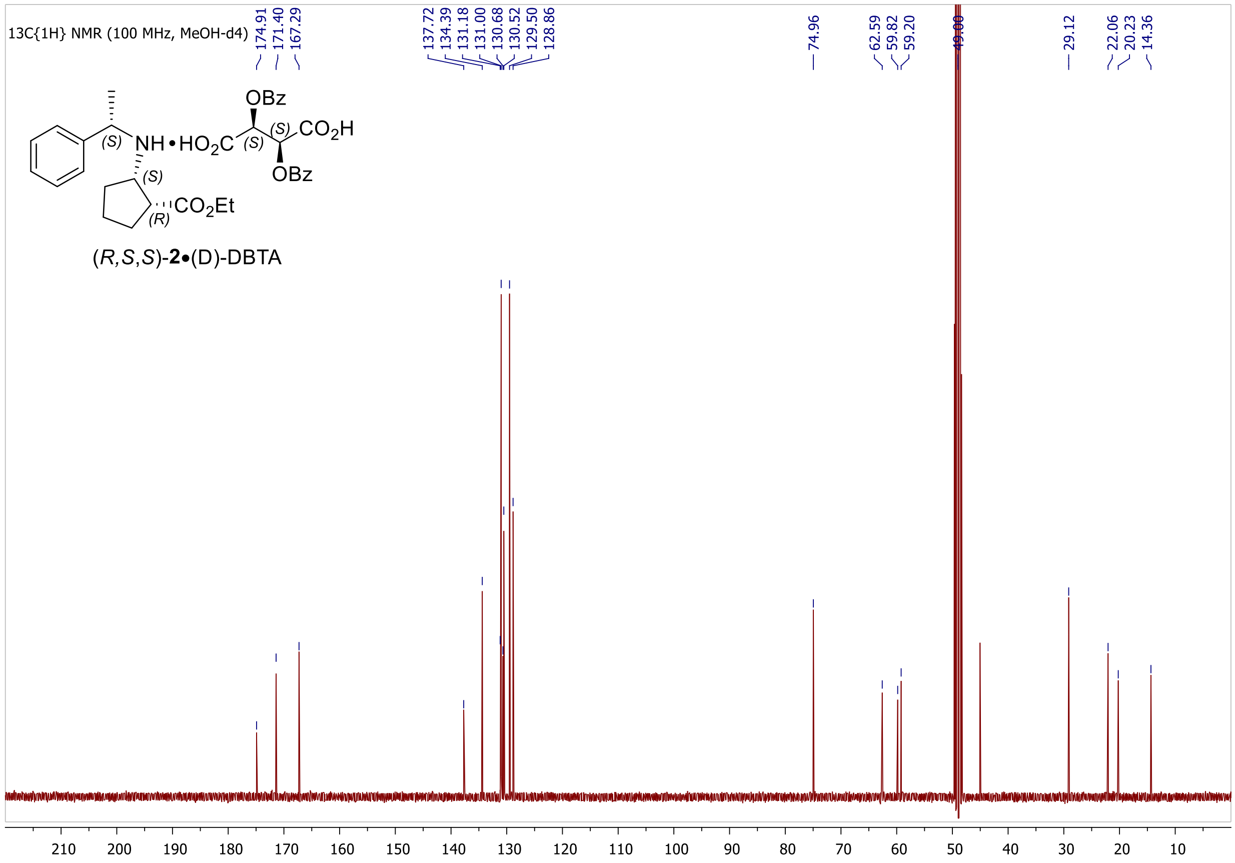
13C{1H} NMR (100 MHz, MeOH-d4)
(R,S,S)-2•(D)-DBTA
174.91 171.40 167.29
137.72 134.39 131.18 131.00 130.68 130.52 129.50 128.86
74.96
62.59 59.82 59.20
49.00
29.12
22.06 20.23 14.36

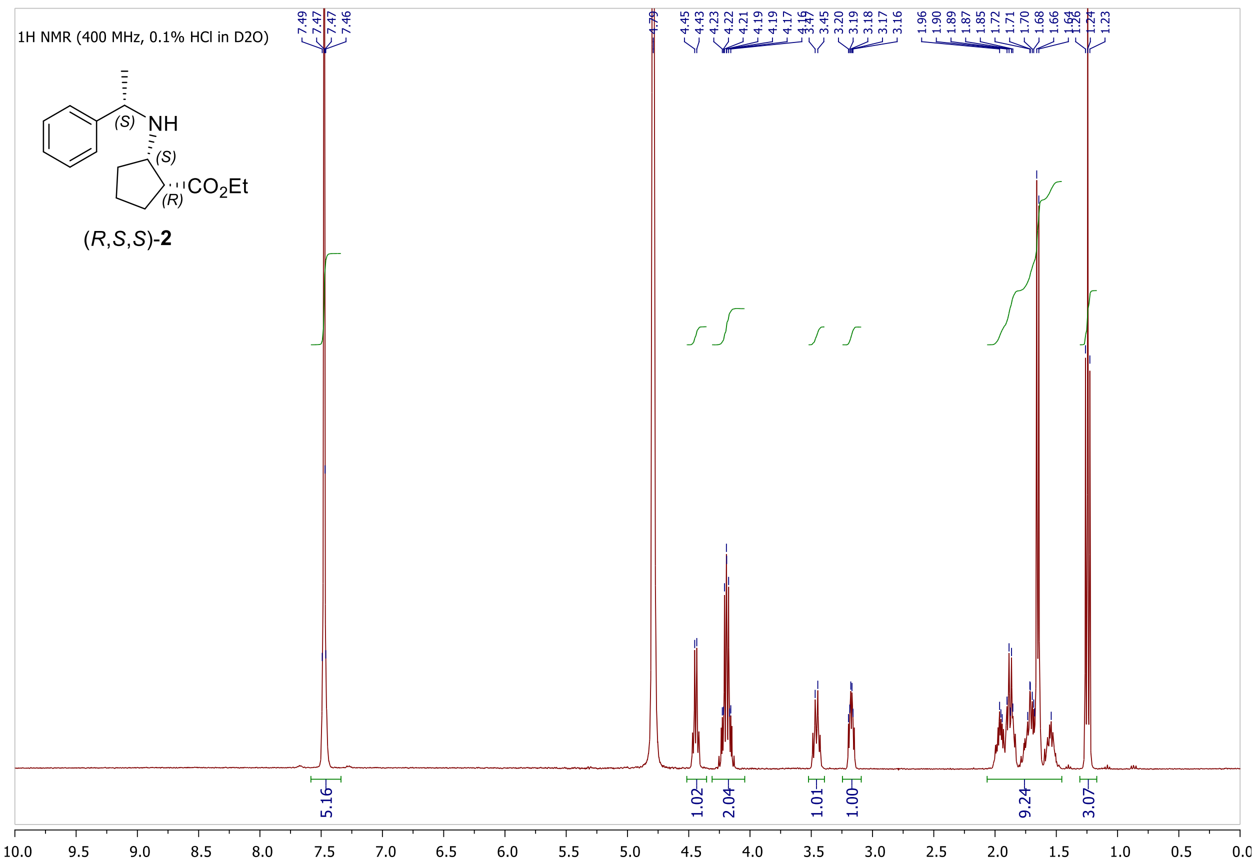
1H NMR (400 MHz, 0.1% HCl in D2O)
(S)
NH
(S)
CO2Et
(R)
(R,S,S)-2
7.49
7.47
7.47
7.46
4.45
4.43
4.23
4.22
4.21
4.19
4.19
4.19
4.17
3.45
3.20
3.19
3.18
3.17
3.16
1.96
1.90
1.89
1.87
1.85
1.72
1.71
1.70
1.68
1.66
1.23
5.16
1.02
2.04
1.01
1.00
9.24
3.07
10.0 9.5 9.0 8.5 8.0 7.5 7.0 6.5 6.0 5.5 5.0 4.5 4.0 3.5 3.0 2.5 2.0 1.5 1.0 0.5

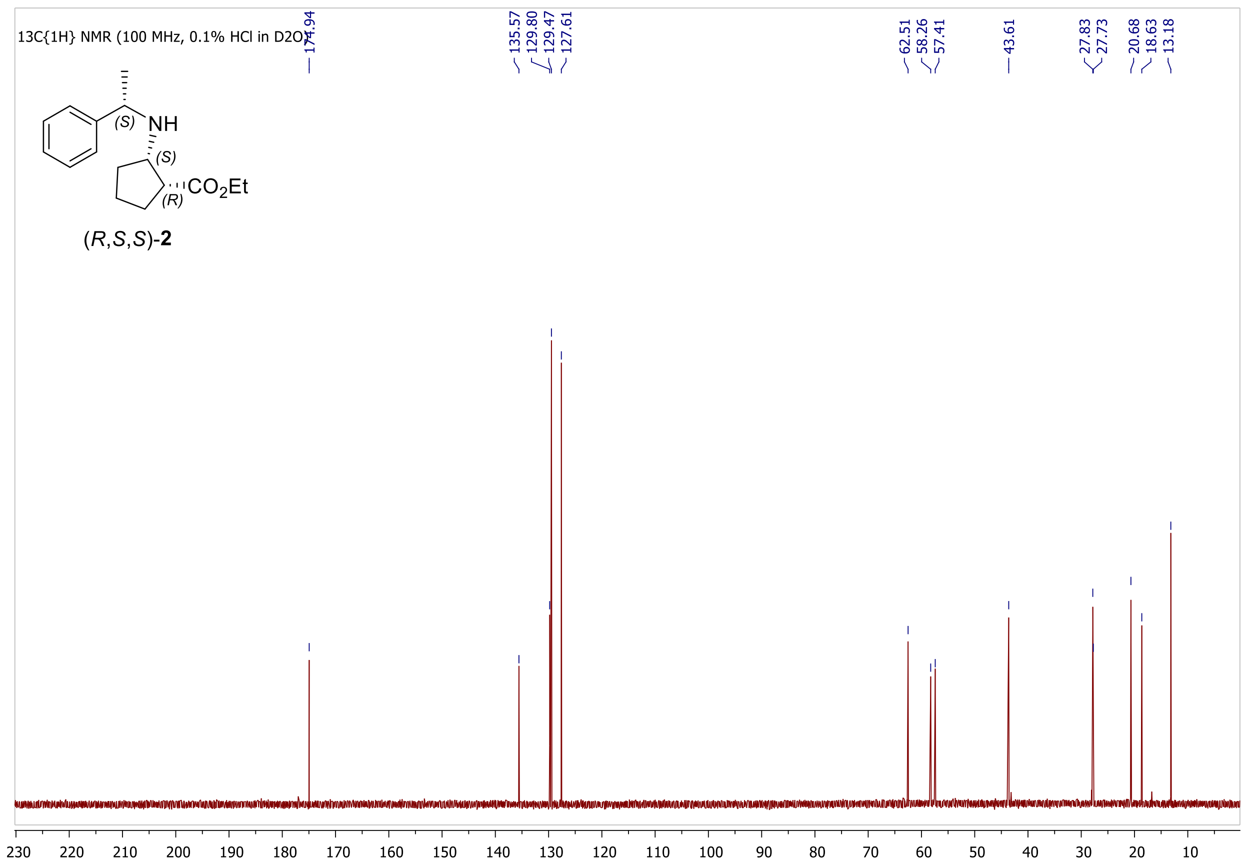
13C{1H} NMR (100 MHz, 0.1% HCl in D2O)
(S)
NH
(S)
CO2Et
(R)
(R,S,S)-2
135.57
129.80
129.47
127.61
62.51
58.26
57.41
43.61
27.83
27.73
20.68
18.63
13.18
230 220 210 200 190 180 170 160 150 140 130 120 110 100 90 80 70 60 50 40 30 20 10

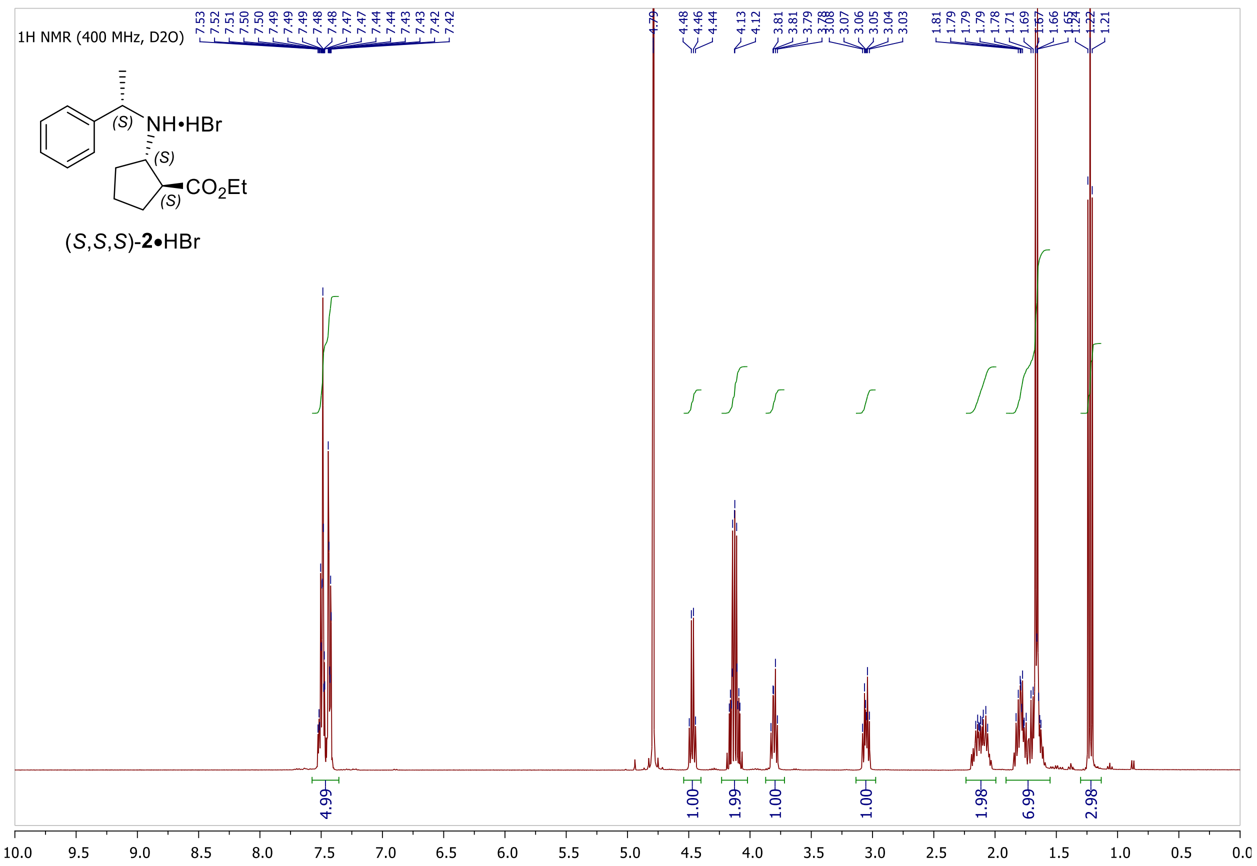

1H NMR (400 MHz, D2O)
(S,S,S)-2•HBr
NH•HBr
CO2Et

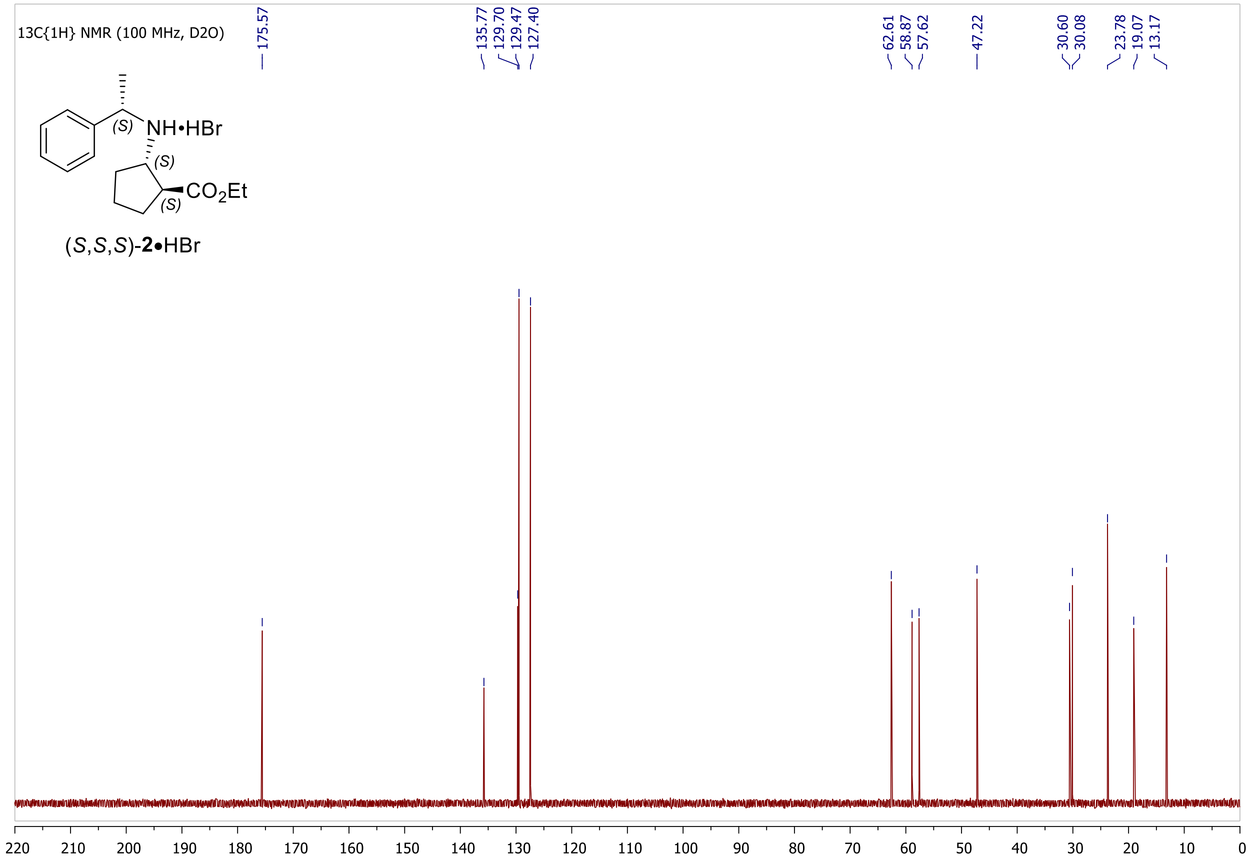

13C{1H} NMR (100 MHz, D2O)
(S,S,S)-2•HBr
NH•HBr
CO2Et
175.57
135.77
129.70
129.47
127.40
62.61
58.87
57.62
47.22
30.60
30.08
23.78
19.07
13.17

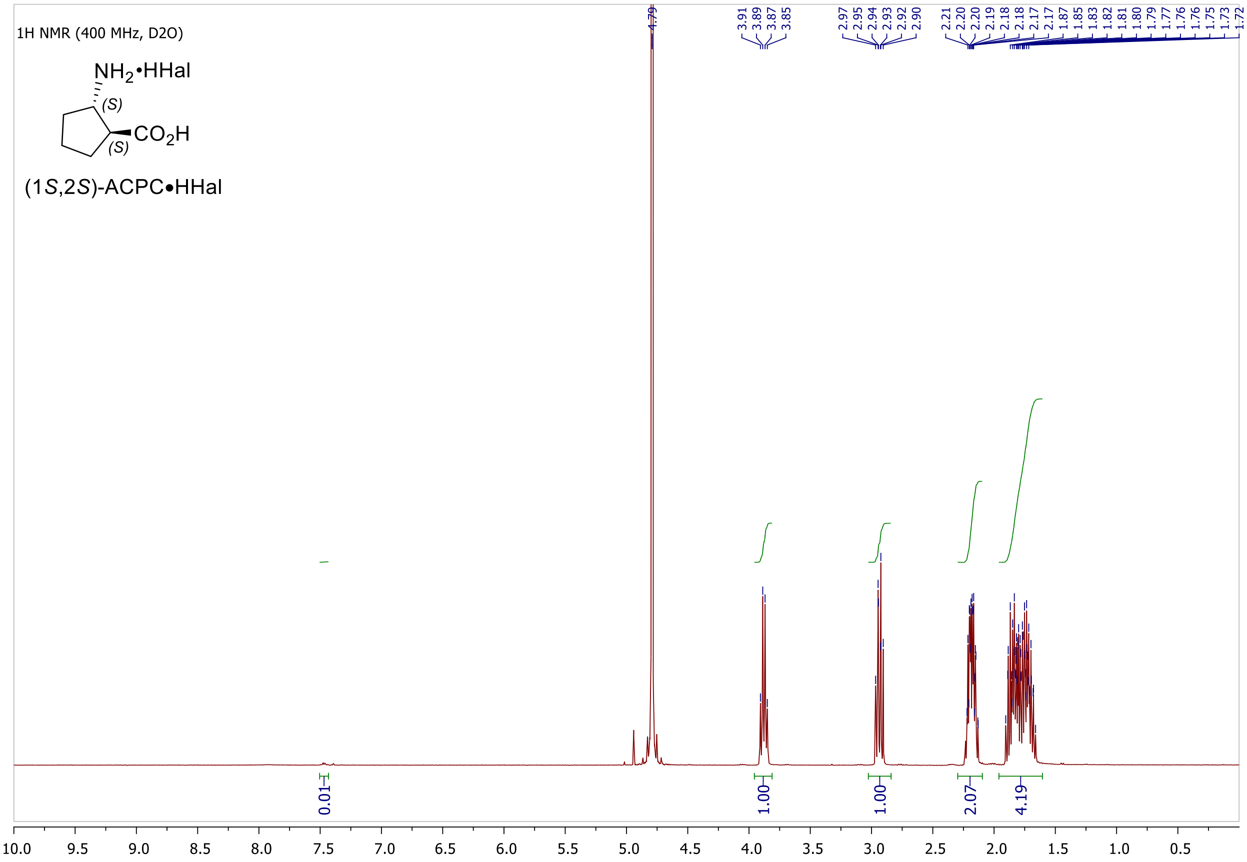
1H NMR (400 MHz, D2O)
NH2•HHal
(S)
(S)
CO2H
(1S,2S)-ACPC•HHal
4.79
3.91
3.89
3.87
3.85
2.97
2.95
2.94
2.93
2.92
2.90
2.21
2.20
2.20
2.19
2.18
2.18
2.17
2.17
1.87
1.85
1.83
1.82
1.81
1.80
1.79
1.77
1.76
1.75
1.73
1.72
0.01
1.00
1.00
2.07
4.19
10.0 9.5 9.0 8.5 8.0 7.5 7.0 6.5 6.0 5.5 5.0 4.5 4.0 3.5 3.0 2.5 2.0 1.5 1.0 0.5

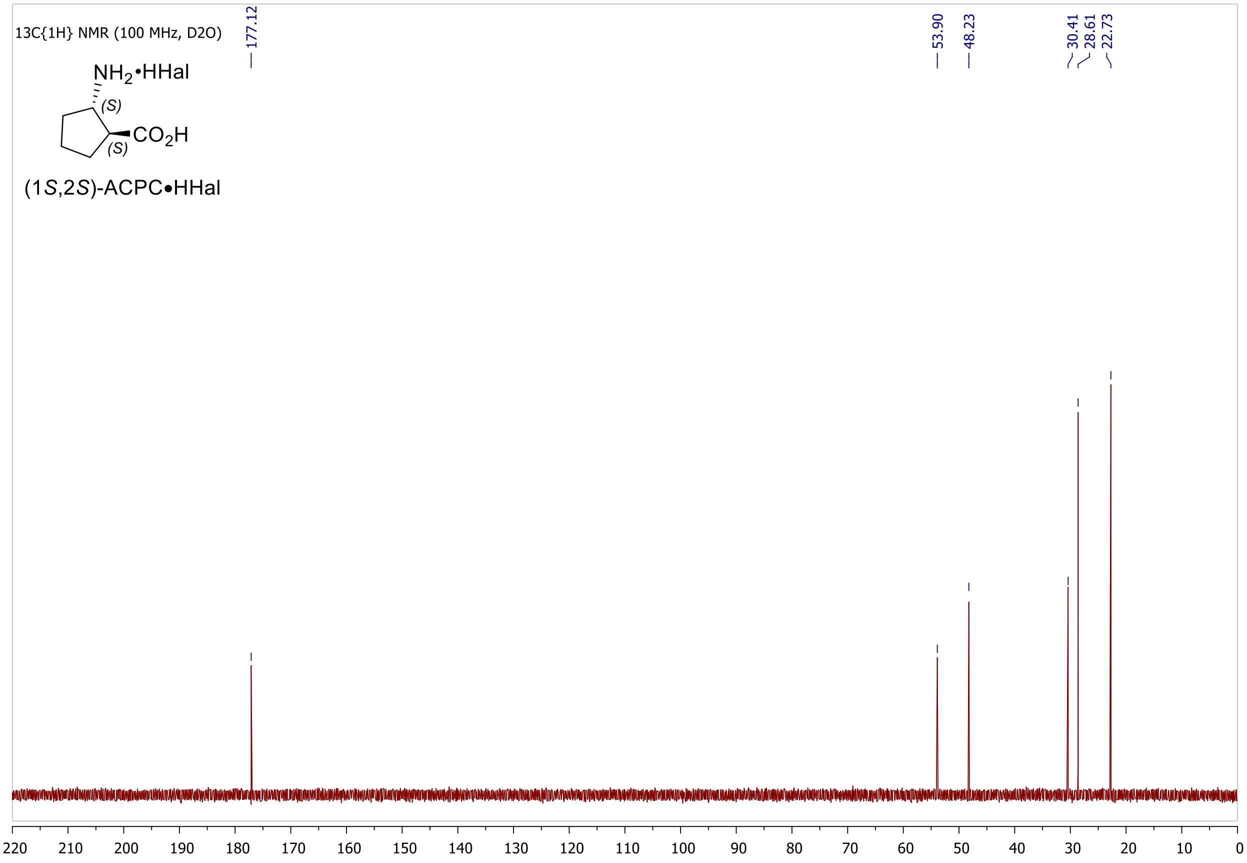
13C{1H} NMR (100 MHz, D2O)
NH2•HHal
(S)
(S)
CO2H
(1S,2S)-ACPC•HHal
177.12
53.90
48.23
30.41
28.61
22.73
220 210 200 190 180 170 160 150 140 130 120 110 100 90 80 70 60 50 40 30 20 10 0

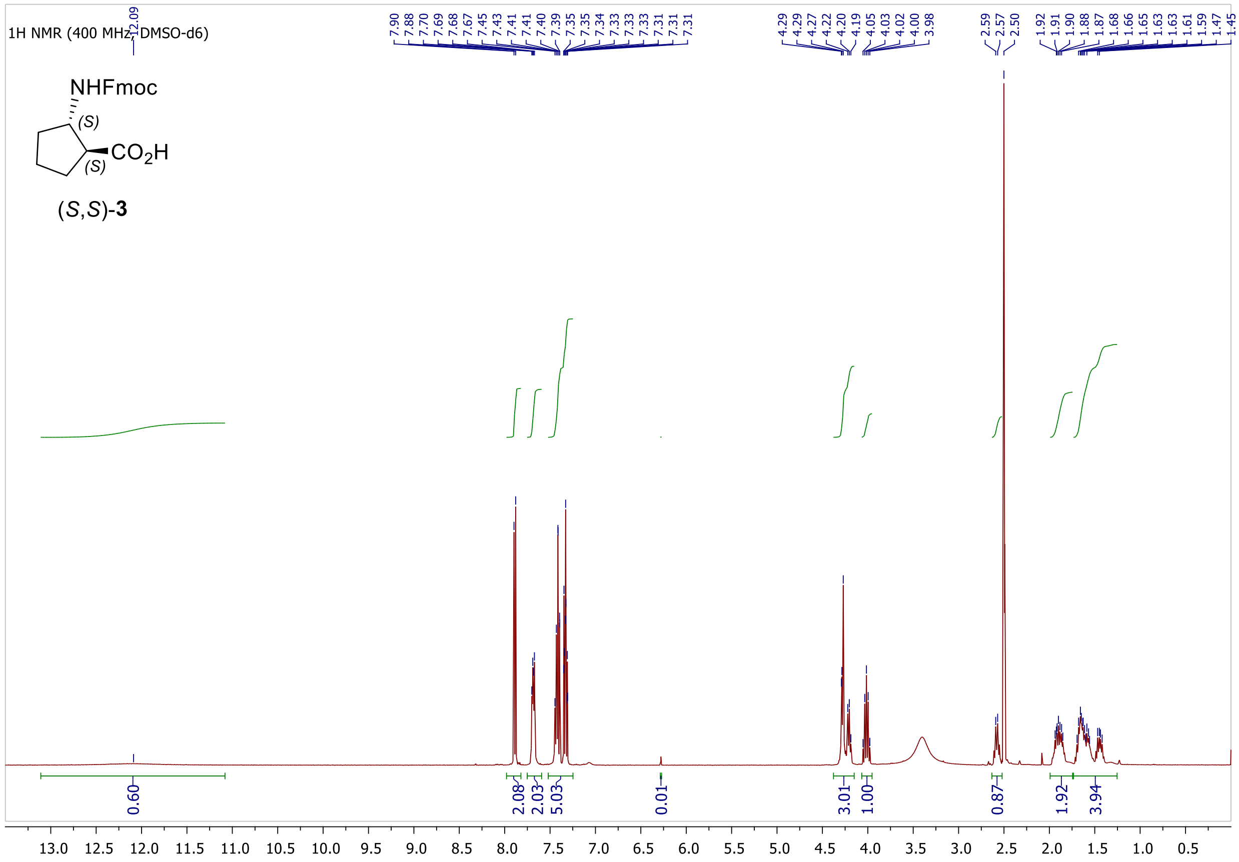
1H NMR (400 MHz, DMSO-d6)
12.09
7.90 7.88 7.70 7.69 7.68 7.67 7.45 7.43 7.41 7.41 7.40 7.39 7.35 7.35 7.34 7.33 7.33 7.33 7.31 7.31 7.31
4.29 4.29 4.27 4.22 4.20 4.19 4.05 4.03 4.02 4.00 3.98
2.59 2.57 2.50
1.92 1.91 1.90 1.88 1.87 1.68 1.66 1.65 1.63 1.63 1.61 1.59 1.47 1.45
NHFmoc
(S)
CO2H
(S)
(S,S)-3
0.60
2.08 2.03 5.03
0.01
3.01 1.00
0.87
1.92 3.94
13.0 12.5 12.0 11.5 11.0 10.5 10.0 9.5 9.0 8.5 8.0 7.5 7.0 6.5 6.0 5.5 5.0 4.5 4.0 3.5 3.0 2.5 2.0 1.5 1.0 0.5

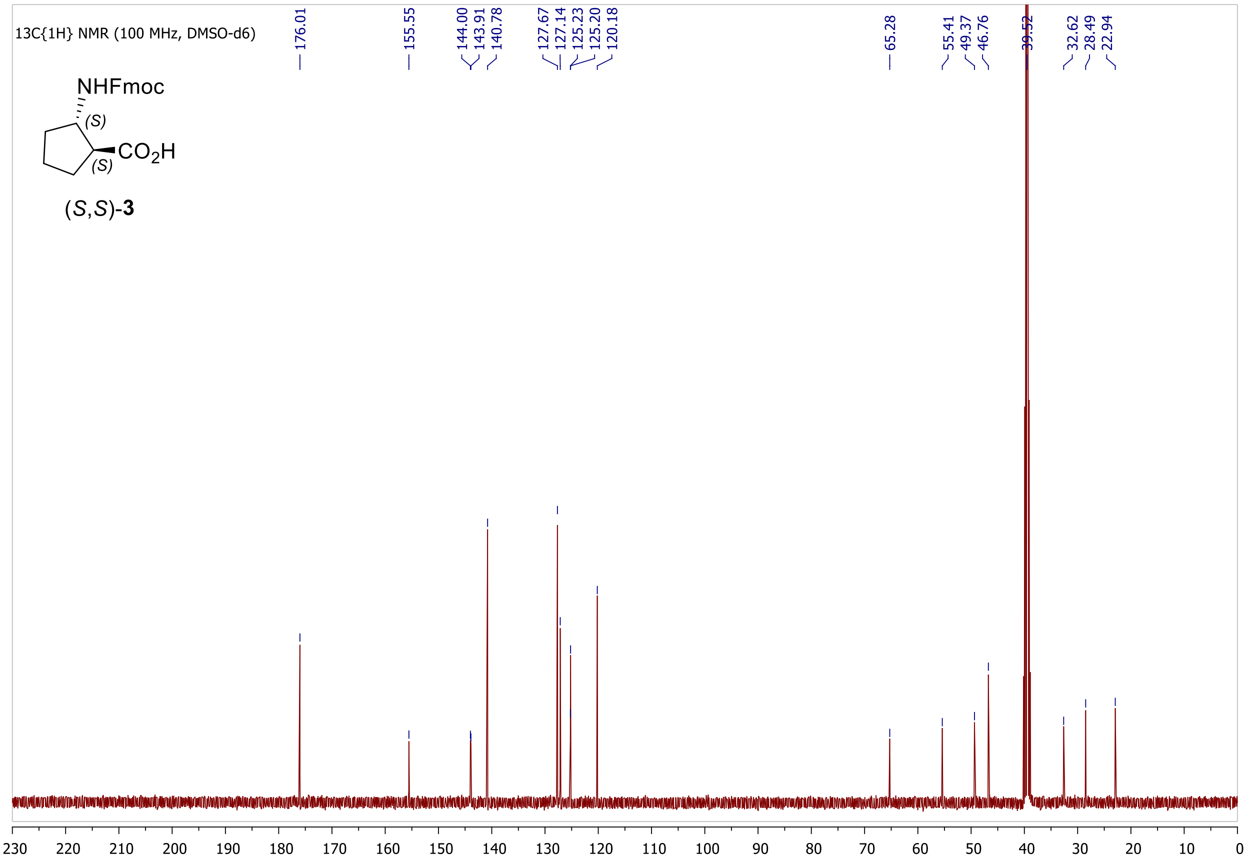
13C{1H} NMR (100 MHz, DMSO-d6)
176.01
155.55
144.00 143.91 140.78
127.67 127.14 125.23 125.20 120.18
65.28
55.41 49.37 46.76
39.52
32.62 28.49 22.94
NHFmoc
(S)
CO2H
(S)
(S,S)-3
230 220 210 200 190 180 170 160 150 140 130 120 110 100 90 80 70 60 50 40 30 20 10 0

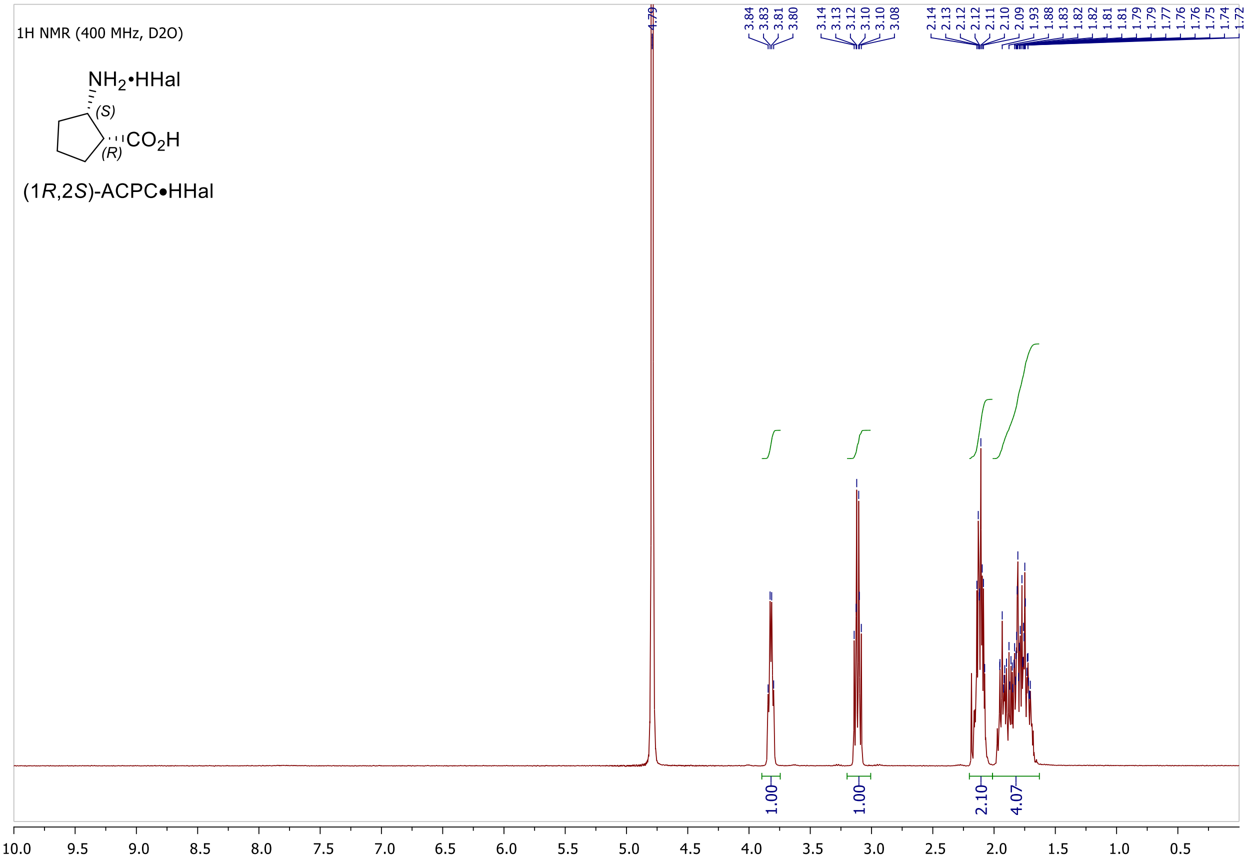
1H NMR (400 MHz, D2O)
NH2•HHal
(S)
(R)
CO2H
(1R,2S)-ACPC•HHal
1.00
1.00
2.10
4.07
10.0 9.5 9.0 8.5 8.0 7.5 7.0 6.5 6.0 5.5 5.0 4.5 4.0 3.5 3.0 2.5 2.0 1.5 1.0 0.5

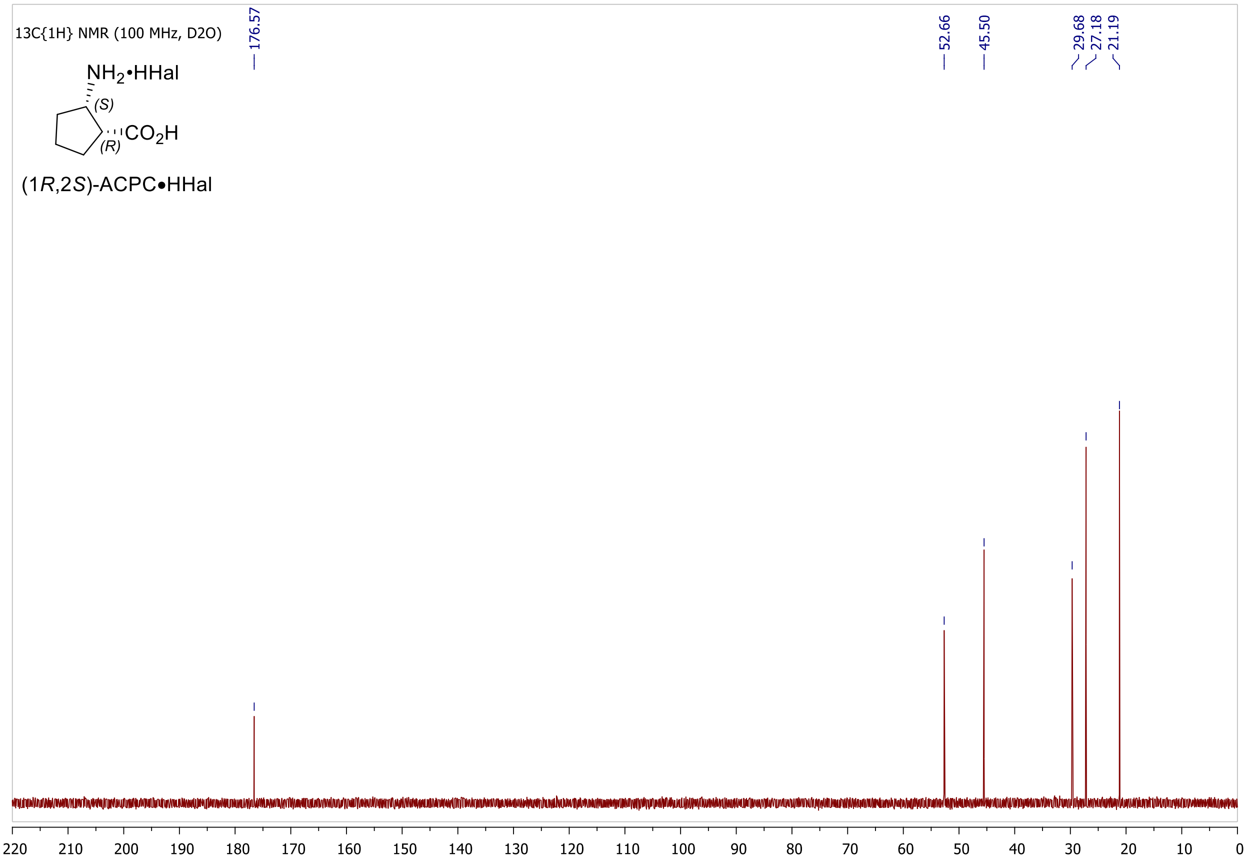
13C{1H} NMR (100 MHz, D2O)
NH2•HHal
(S)
(R)
CO2H
(1R,2S)-ACPC•HHal
176.57
52.66
45.50
29.68
27.18
21.19
220 210 200 190 180 170 160 150 140 130 120 110 100 90 80 70 60 50 40 30 20 10 0

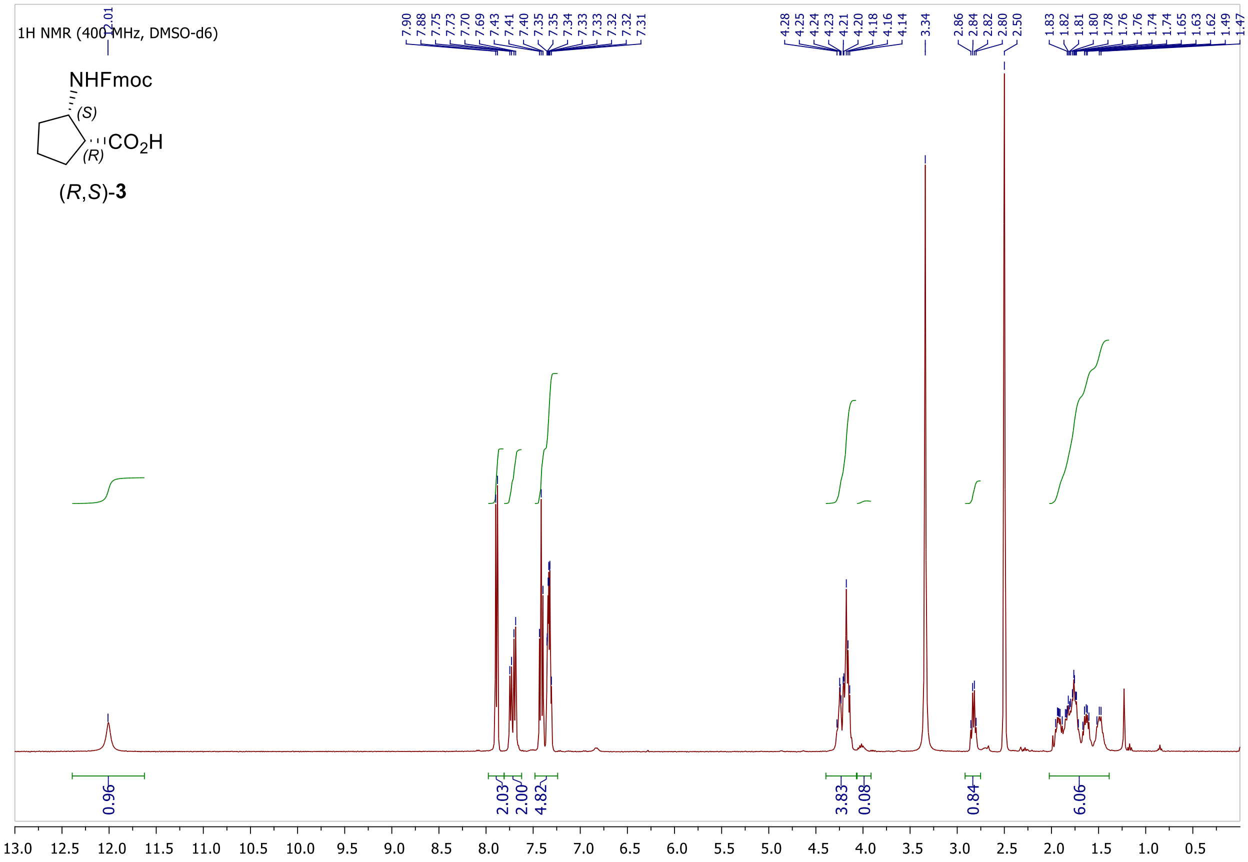
1H NMR (400 MHz, DMSO-d6)
NHFmoc
(S)
CO2H
(R)
(R,S)-3
12.01
7.90 7.88 7.75 7.73 7.70 7.69 7.43 7.41 7.40 7.35 7.34 7.33 7.33 7.32 7.32 7.31
4.28 4.25 4.24 4.23 4.21 4.20 4.18 4.16 4.14
3.34
2.86 2.84 2.82 2.80 2.50
1.83 1.82 1.81 1.80 1.78 1.76 1.76 1.74 1.74 1.65 1.63 1.62 1.49 1.47
0.96 2.03 2.00 4.82 3.83 0.08 0.84 6.06
13.0 12.5 12.0 11.5 11.0 10.5 10.0 9.5 9.0 8.5 8.0 7.5 7.0 6.5 6.0 5.5 5.0 4.5 4.0 3.5 3.0 2.5 2.0 1.5 1.0 0.5

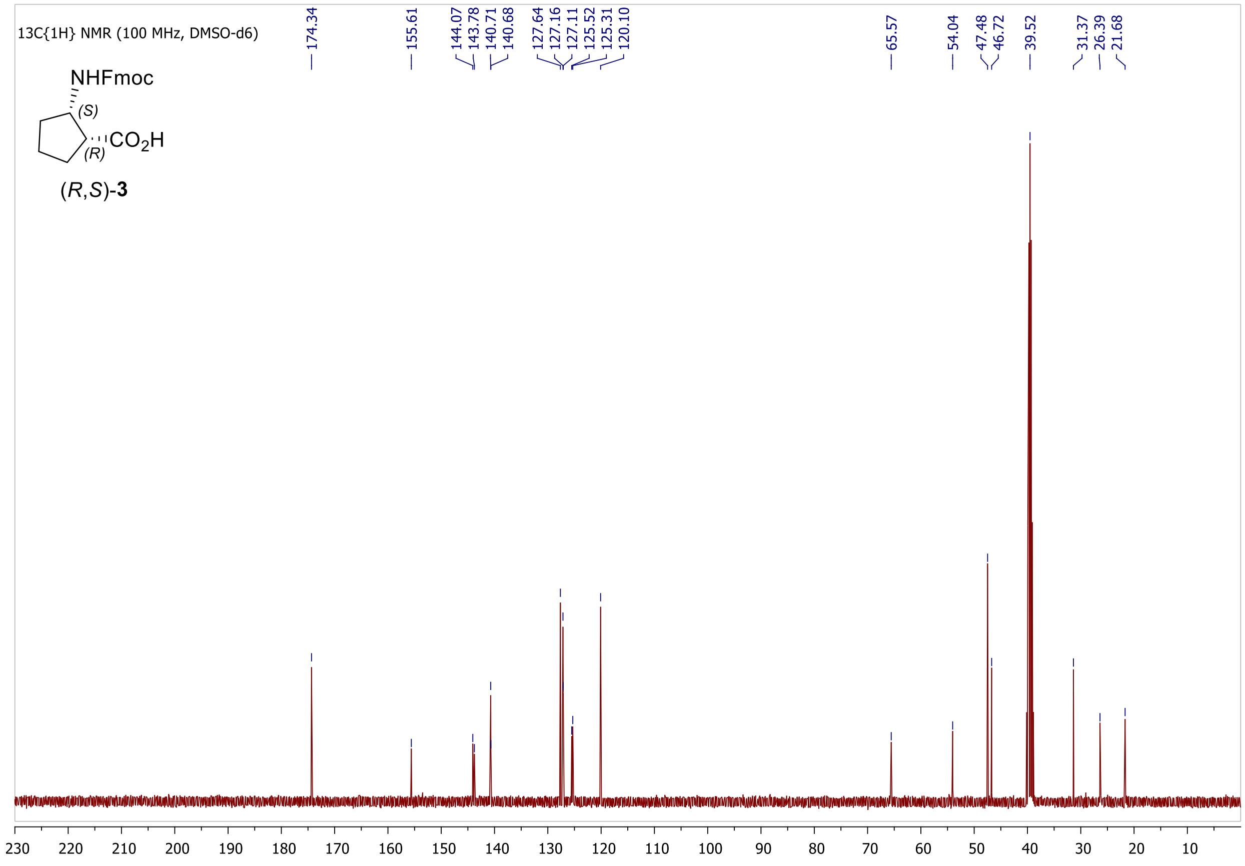
13C{1H} NMR (100 MHz, DMSO-d6)
NHFmoc
(S)
CO2H
(R)
(R,S)-3
174.34
155.61
144.07 143.78 140.71 140.68
127.64 127.16 127.11 125.52 125.31 120.10
65.57
54.04 47.48 46.72 39.52
31.37 26.39 21.68
230 220 210 200 190 180 170 160 150 140 130 120 110 100 90 80 70 60 50 40 30 20 10

References

[1] Rigaku Oxford Diffraction, *CrysAlisPRO* Software system, Oxford, UK, 2022.

[2] O. V. Dolomanov, L. J. Bourhis, R. J. Gildea, J. A. Howard, H. Puschmann, *J. Appl. Crystallogr.*, 2009, **42**, 339-341.

[3] G.M. Sheldrick, *Acta Crystallogr., Sect. A*, 2015, **A71**, 3-8.

[4] G.M. Sheldrick, *Acta Crystallogr., Sect. C*, 2015, **C71**, 3-8.

[5] S. Parsons, H. D. Flack, T. Wagner, *Acta Crystallogr., Sect. B*, 2013, **B69**, 249-259.

[6] Hee-Seung Lee, Kim Danim, Jaewook Kim, *CSD Communication (Private Communication)* 2021

[7] K. Brandenburg, DIAMOND, Version 4.6.8. Crystal Impact GbR, Bonn, Germany, 2022
